# Residue Interaction Network Reveals Allosteric Pathways Linking Orthosteric and Intracellular Sites in Class A GPCRs

**DOI:** 10.1021/acs.jcim.6c00526

**Published:** 2026-08-20

**Authors:** Sonja Peter, Georges Chalhoub, Peter J. McCormick, Chris De Graaf, Ijen Chen, Giovanni Bottegoni

**Affiliations:** † Nxera Pharma U.K., Cambridge CB21 6DG, U.K.; ‡ Department of Biomolecular Sciences, University of Urbino Carlo Bo, Urbino 61029, Italy; § Institute of Systems, Molecular & Integrative Biology, University of Liverpool, Liverpool L69 7ZX, U.K.; ∥ Structure Therapeutics, San Francisco, California 94080, United States; ⊥ Department of Pharmacy, University of Birmingham, Birmingham B15 2TT, U.K.

## Abstract

Understanding how allosteric modulators influence protein dynamics is essential for guiding drug design. This work analyses a total of 45 μs of classical molecular dynamics simulations for four class A G-protein-coupled receptors (GPCRs), namely the Complement C5a receptor (C5AR1), the Purinergic Receptor P2Y (P2RY1), and the Cannabinoid Receptors 1 and 2 (CNR1/CNR2). Protein dynamics is essential to detect the shallow extrahelical binding sites, such as the one found in P2RY1. Current methods for computing Allosteric Communication Networks (ACNs) produce complex outputs requiring expert interpretation. To address this, we focus on the shortest paths of information transfer between the orthosteric and G-protein binding sites in Class A GPCRs. Our retrospective analysis reveals state- and bias ligand-dependent residue interactions along these communication pathways. Furthermore, focusing on the predicted binding site of allosteric modulator EC21a at cannabinoid receptors, the ACN framework was used to prioritize two residues for mutational analysis that may contribute to allosteric communication.

## Graphical abstract

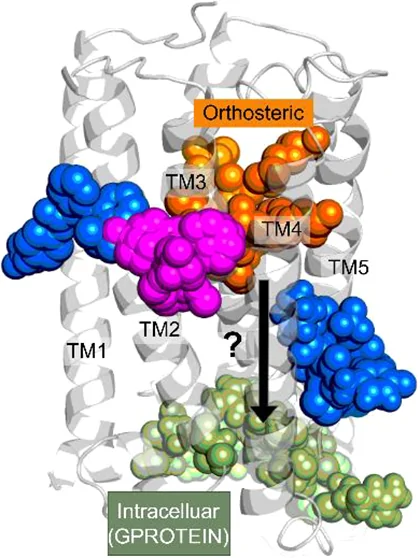

## Introduction

Mediating the communication between the cell and the extracellular environment, G protein-coupled receptors (GPCRs) are involved in almost all physiological processes.^1^ Their role in signal transduction is predicated on the structural plasticity that the members of this protein family possess. GPCRs exist in a thermodynamic equilibrium, constantly interconverting between different conformational states. This equilibrium can be modulated by various factors, such as the binding of endogenous ligands at orthosteric or allosteric sites, interactions with other proteins, or regulatory enzyme activity (e.g., kinases). Additionally, the receptor’s conformational ensemble can be influenced by genetic mutations or variations in the membrane composition. Because of their physiological role, GPCRs are the target of over one-third of all marketed drugs.^2^ Small organic molecules and synthetic peptides exert their effects by stabilizing specific functional states of G protein–coupled receptors (GPCRs). The growing availability of high-resolution GPCR structures has greatly advanced our understanding of the conformational landscapes underlying receptor activation and signaling. This insight has, in turn, strengthened rational drug design, making structure-based approaches an increasingly common strategy for developing novel ligands. Importantly, the expanding structural repertoire now includes numerous complexes with allosteric modulators, highlighting the significance of sites beyond the orthosteric pocket.^3^ However, GPCRs are inherently dynamic, and static structures offer only a partial view of their conformational landscape. A comprehensive understanding therefore requires complementary dynamic approaches. Beyond static structures, molecular dynamics simulations can reveal how ligands influence receptor flexibility. This is particularly relevant for allosteric modulators, whose binding often induces subtle, long-range changes in receptor dynamics that may not be fully captured by static structures but can critically affect functional states and interhelical communication.

The strategy of describing proteins as networks, where residues are represented as nodes and their interactions (e.g., spatial proximity, electrostatic coupling, torsional correlations, or atomic displacement) as edges, has been successfully applied to GPCRs to investigate allosteric communication, identify functional sites, and support drug design.^4^ The mechanistic study published in 2021 by Hauser et al.^5^ described interaction networks based on static structures by comparing state-specific contacts between active and inactive GPCR conformations. In Class A GPCRs, 32 state-dependent contacts were identified, with 75% occurring in the inactive state and 25% in the active state. In another study by Yadav et al.,^6^ machine learning approaches, including XGBoost, 3D convolutional neural networks, and graph neural networks, were employed to classify GPCR activation states as active, intermediate or inactive. XGBoost achieved the highest predictive accuracy when trained on 105 selected residue pairs. Notably, residue pair distances between generic positions 3.40–7.49, 6.38–7.46, 6.31–3.32, and 7.41–6.34 (the Ballesteros and Weinstein numbering is applied throughout the manuscript) were key contributors to predictive performance. A recent review by Nerin-Fonz et al.^7^ highlights that both AR-Rpred^8^ and NACEN^9^ use network-based features for allosteric site prediction. These methods combine network-based information with physicochemical properties and conservation scores. In addition, AR-RPred incorporates dynamic features derived from normal-mode analysis. The resulting feature sets are used to train random forest models. AR-RPred achieves an accuracy of 70% and a recall of 80%, while NACEN shows a higher accuracy of 83% but a much lower recall of 30%. This comparison suggests that incorporating dynamic features can significantly enhance the predictive performance for identifying allosteric binding sites. AlloSigma^10,11^ calculates the per residue change in free energy based on ligand binding or mutation, enabling mechanistic studies of allosteric sites. The rigidity transmission allostery algorithm^12,13^ predicts how a mechanical perturbation of rigidity, such as that induced by ligand binding at one site, propagates through the protein network, leading to altered conformational degrees of freedom at a distant site.

The combination of MD simulations with residue interaction networks and shortest path analysis between pairs of nodes, usually called source and target, offers a comprehensive view of how residues communicate and interact over time.^14^ Quantification of residue–residue interactions based on interaction frequency or contact probability has been widely used to construct and weight such networks,^15,16^ enabling the identification of persistent and transient communication pathways across conformational ensembles in enzymes,^17–19^ GPCRs^20^ and kinases.^21^ This dynamic perspective also captures transient interactions that are often hidden in static representations. For instance, Ferraro et al.^22^ analyzed MD trajectories with clustering and dihedral principal component analysis to investigate how local structural variations in a compound series influence the efficacy of dopamine D3 receptor modulators. A study investigating the inactive, intermediate and active structures of the β_2_-adrenergic receptor (ADRB2) analyzed correlated torsional angle movements from microsecond-scale molecular dynamics simulations and applied shortest-path calculations to identify allosteric pathways connecting the extracellular and intracellular GPCR binding sites. Notably, in the inactive state, these pathways were more localized in transmembrane helix 6 (TM6), but shifted toward TM7 in the fully active conformation, indicating a dynamic redistribution of allosteric signaling during receptor activation. Additionally, mutations that alter ligand efficacy without affecting binding affinity were found to be more frequently located within these allosteric pathways.^23^ Morales-Pastor et al.^24^ conducted protein network analysis and alanine-scanning mutagenesis on 360 residues to investigate biased signaling in Cannabinoid Receptor 2 (CNR2). They found that Gαi2 coupling is more resilient to mutations than β-arrestin1, a consistently observed trend for class A GPCRs. In line with the results obtained for ADRB2, they found that inactive states bound to inverse agonists primarily use TM6 for communication, while agonist-bound active states favor TM7. Gαi_2_ preferential mutants cluster near high-transmission nodes and exhibit greater network connectivity. Notably, different mutations could produce the same functional outcome (e.g., enhanced Gαi_2_ coupling) and disruption of conserved motifs such as DRY, NPxxY, toggle switch or sodium binding site impaired β-arrestin recruitment. The integration of molecular dynamics and network analysis can now be fully leveraged thanks to several open-source Python tools, including MDpath^25^ for dihedral movement analysis, BaNDyT^26^ for probabilistic network modeling, MDigest^27^ and AlloViz^28^ for protein contact correlation and visualization, enabling a detailed exploration of allosteric communication pathways.

Allosteric communication networks (ACNs) are increasingly used to characterize signal transmission within GPCRs. With Class A GPCRs in mind, we therefore examined whether focusing on the identification and characterization of the shortest paths connecting the orthosteric binding site to the intracellular region, where the G protein binds, could effectively capture key routes of allosteric signal transmission. For this study, we chose representative receptors from three subfamilies: the Complement C5a Receptor (C5AR1) from the Peptide subfamily, the Purinergic Receptor 2Y (P2RY1) from the Nucleotide subfamily, and the Cannabinoid Receptors 1 and 2 (CNR1 and CNR2) from the Lipid subfamily (Figure 1). We selected allosteric modulators known to influence the transmembrane region, excluding those that act primarily through steric hindrance, such as by blocking G-protein binding, as seen in chemokine receptors, or by stabilizing the ICL2 loop to promote G-protein coupling, as observed in dopamine and fatty acid receptors. The results obtained from the investigated systems suggest that this technique can serve as an effective approach, providing both retrospective mechanistic insight and may also support hypothesis generation in drug discovery. More broadly, the framework may be transferable to other members of the Class A GPCR family, although broader validation will be required to assess its general applicability.

**Figure 1:**
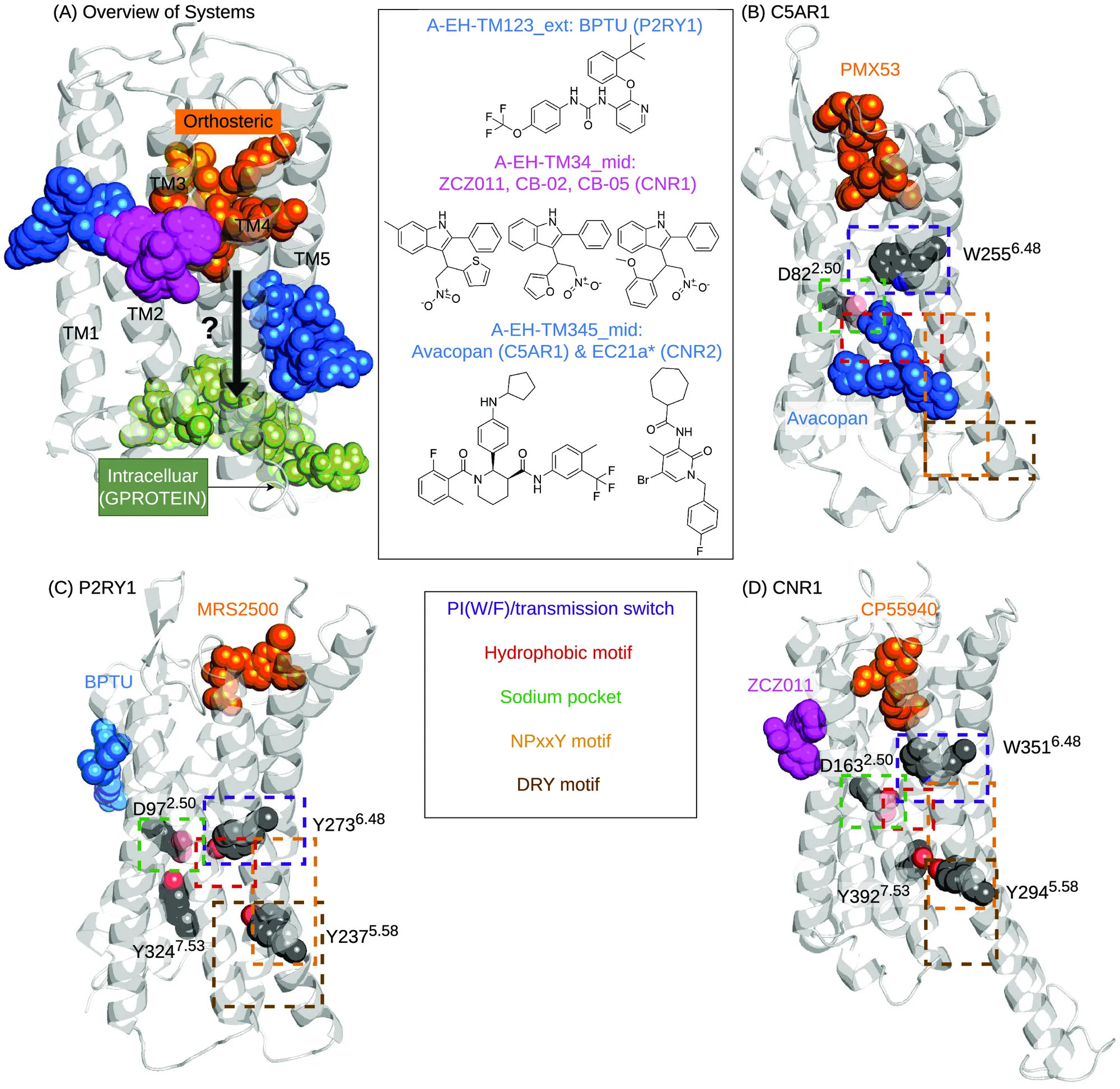
Binding sites of allosteric modulators relative to state-specific contacts reported by Hauser et al.^5^ (A) Binding sites of extrahelical allosteric modulators are shown on the Cannabinoid Receptor 1 structure (PDB: 7WV9), with inactivating modulators in blue and activating modulators in magenta. Orthosteric-site residues are shown as orange spheres and intracellular-site residues in green. The 2D structures of BPTU, ZCZ011, CB-02, CB-05, Avacopan, and EC21a are displayed; the EC21a pose is based on the docking model reported by Farooq et al.^29^ Conserved microswitches are shown for C5AR1, P2RY1, and CNR1.(B–D) Binding sites of Avacopan (B), BPTU (C), and ZCZ011 (D) relative to key Class A GPCR activation microswitches, including the CWxP/transmission switch (purple), the hydrophobic motif (dark red), the sodium pocket (light green), the NPxxY motif (orange), and the DRY motif (brown).

## Methods

### Structure Preparation

MD simulations were performed using refined, high-resolution crystal structures obtained from GPCRdb^30–33^ as reported in Table 1. The C- and N-terminus were trimmed according to the structurally resolved residues in the corresponding experimental structure. For C5AR1 (PDB: 7Y66), P2RY1 (PDB: 4XNV and 8WJX), CNR1­(PDB: 7WV9 and 6KPG), and CNR2 (PDB: 8GUS) the refined model was not available on GPCRdb.^30–33^ A reference structure in the same conformational state or GPCRdb^30–33^ α Fold model bound to Gio was used to rebuild the loops using Schrödinger Suite multiple templates (chimera) protocol as indicated in Table 1. Protein preparation was carried out in the Schrödinger Suite. Disulfide bonds were assigned, residue p*K*_a_ values were predicted with PROPKA at pH 7.4, and protonation states were set accordingly before hydrogen atoms were added. Asp^2.50^ was protonated in the active-state structures. The hydrogen-bonding network was then optimized, including sampling of the orientations of retained water molecules. Finally, the structures were energy-minimized with the OPLS4 ^34^ force field to an RMSD convergence threshold of 0.3 Å. For the ligands, the most populated protonation state of titratable atoms at pH 7.4 ± 1.0 was assigned using Epik. All protein chains other than the receptor and, for the active state structures, the G-protein α subunit were deleted. Co-crystallized waters and the sodium ion in the sodium pocket were retained. All small molecules except the orthosteric and/or allosteric ligands (Table 1) were deleted.

**Table 1:** Case Studies for MD Simulation Allosteric Communication Network Investigation including Protein Families; Complement C5a Receptor (C5AR1), the Purinergic Receptor P2Y (P2RY1), and the Cannabinoid Receptor 1 and 2 (CNR1/CNR2), Conformation, Structure ID, Mutations, Protonation and Ligands either Allosteric (a) or Orthosteric (o)^a^

Protein Family	Conformation	PDB	Resolution (Å)	Reference (PDB ID) for ICL3	Ligand (mode of action)
C5AR1	Inactive	6C1R	2.2	-	a: Avacopan (allosteric antagonist)o: PMX53peptide (antagonist)
	Inactive	6C1R	2.2	-	o: PMX53peptide (antagonist)
	Active	7Y66	2.9	7Y67	o: BM213 peptide (agonist)
P2RY1	Intermediate	4XNV	2.2	4XNW	a: BPTU (allosteric antagonist)
	Intermediate	4XNW	2.7	-	o: MRS2500 (antagonist)
	Active	8WJX	3.2	7XXH	o: ADP (agonist)
CNR1	Inactive	5U09	2.6	-	o: Taranabant (antagonist)
	Active	7WV9	3.4	GPCRdb^30–33^ α Fold model bound to Gio	a: ZCZ011 (positive allosteric modulator)o: CP55940 (agonist)
	Active	8IKG	3.4	-	a:3-[(1*S*)-1-(furan-2-yl)-2-nitro-ethyl]-2-phenyl-1*H*-indole (allosteric agonist)
	Active	8IKH	3.3	-	a:3-[(1*R*)-1-(2-methoxyphenyl)-2-nitro-ethyl]-2-phenyl-1*H*-indole (allosteric agonist)
	Active	6KPG	3.0	GPCRdb^30–33^ α Fold model bound to Gio	o: AM12033 (agonist)
CNR2	Inactive	5ZTY	2.8	-	o: AM10257 (antagonist)
	Active	8GUR	2.8	-	o: CP55940 (agonist)
	Active	8GUS	3.0	8GUR	o: HU803 (agonist)

a Each MD simulation was performed in triplicate (1 μs each).

### Molecular Dynamics Simulations

MD simulations were initiated from equilibrated protein–membrane systems. Ligand parameters were evaluated with the Force Field Builder module in Schrödinger release 2023–04 (Schrödinger, New York, NY, USA) to determine whether high-quality OPLS4 torsional parameters were available. When required, missing dihedral parameters were generated using the standard Force Field Builder workflow, which, according to documentation, includes fragmentation of the ligand, identification of torsions not covered by high-quality parameter patterns, torsional scans, and fitting of torsional terms to reproduce the corresponding reference energy profiles.^35^ For this procedure, we used the QRNN-TB method, which combines a charge recursive neural network potential with extended tight binding.^36^ The fitted torsional parameters were then incorporated into the OPLS4 force field for subsequent simulations.^34^ The simulations were carried out in an orthorhombic box extending 20 Å beyond the solute. In the first step, the structures were embedded in a 1-palmitoyl-2-oleoly phosphatidylcholine (POPC: 300 K) membrane bilayer using the orientation from the Orientations of Proteins in Membranes (OPM) database^37^ as reference. The generated complex was solvated with TIP3P waters. Counterions were used to neutralize the box. In the second step, the systems were minimized for 100 ps. In a third step, the systems were equilibrated for 10 ns using the constant surface tension (NPγT) ensemble and the standard Schrödinger protocol was used for equilibration and thermalization of the membrane. For the inactive-, intermediate-, and active-state structures lacking a G-protein α-subunit, unconstrained MD simulations were performed for 1 μs, with snapshots saved every 200 ps. Simulations were carried out in the NPγT ensemble, with the surface tension set to 0 bar·Å and the pressure maintained at 1.01325 bar using the Martyna-Tobias-Klein barostat (τ_MTK_ = 2.0 ps). The temperature was kept at 300 K with a Nosé-Hoover chain thermostat (τ_NHC_ = 1.0 ps). Short-range interactions were truncated at 9.0 Å, whereas long-range electrostatics were treated with the Particle Mesh Ewald method.^38^ Bond constraints involving hydrogen atoms were treated with the M-SHAKE algorithm.^39^ Integration was performed using RESPA multiple-time-step integrator^40^ with a 2 fs time step, except for long-range electrostatics that were computed every 6 fs (Figures S1–S5). For the active-state structures with a G-protein α-subunit, a harmonic restraint with a force constant of 1 kcal mol^–1^ Å^–2^ on the backbone atoms of the G-protein α-subunit was used. VMD was used to convert the trajectories from a Desmond (-in.cms/.dtr) to Gromacs (.pdb/.trr) format. Then, the.pdb/.trr file was converted to a.gro/.xtc containing only the GPCR protein of interest using *MDanalysis*.^41,42^

### Identification and Analysis of Protein Binding Sites

For each system, binding site analysis was performed with the Pocketron^43^ algorithm (Tables S1–S5) as implemented in BiKi Life Sciences 1.0,^44^ using molecular dynamics trajectories generated from three independent replicas. Static pocket detection was performed on each frame, enabling the evaluation of the evolution of the pocket throughout the simulation. Only pockets with a volume equal or greater than 34.5 Å^3^ (∼3 water molecules) were considered. To enable comparison across receptors, pocket residues were mapped to generic GPCR residue numbers (Ballesteros–Weinstein^45^) via sequence alignment. Binding sites were subsequently annotated using a standardized scheme following the format *Class–Sublocation–ResidueSet–MembranePosition*,^3^ where the sublocation distinguishes extrahelical (EH), intrahelical (IH), and intracellular (IC) sites. For extrahelical pockets, the membrane position (ext, mid, int) was determined from the normalized z-coordinates of pocket residues after alignment to the Orientations of Proteins in Membranes (OPM) reference. As a representative example, the pocket corresponding to the Avacopan binding site in C5AR1 is annotated as A-EH-TM345_mid, indicating a class A receptor pocket located in an extrahelical region, formed by residues from transmembrane helices 3–5, and positioned in the middle of the membrane.

### Calculation of Residue Contacts

The residue interactions from the MD trajectory were calculated using the *GetContacts* python package (github.com/getcontacts/getcontacts) through *AlloViz.* ^28^ The detailed distance and geometric requirements for the different types of interactions including hydrogen bonds, salt bridges, pi-cation, pi-stacking, t-stacking, hydrophobic and van der Waals interactions can be found in the repository. The overall contact frequency was calculated as the fraction of simulation frames in which a contact between two residues was observed, relative to the total number of frames.

### Calculation of Allosteric Communication Network

The Allosteric Communication Network (ACN) was constructed as a graph with nodes corresponding to amino acid residues and edges weighted by residue contact frequencies (range: 0–1). Based on this graph, shortest paths between the class A orthosteric residues 3.32, 3.33, 3.36, 45.52, 5.43, 6.48, 6.51, 6.55, 7.38, 7.42 and any of the intracellular residues 2.39, 3.50, 3.53, 3.54, 3.56, 5.65, 5.68, 6.25, 6.26, 6.29, 6.32, 6.33, 8.47, 8.48 were identified using Dijkstra’s algorithm as implemented in NetworkX (github.com/networkx/networkx). The generic residue numbers were selected based on prior analysis of common class A residues in contact with orthosteric ligands and the G-protein, respectively.^3^ To derive distances suitable for path-based analyses, absolute contact weights were transformed according to

$$d_{\mathrm{ij}}=-\log\left(\left|w_{\mathrm{ij}}\right|+\varepsilon \right)+\varepsilon$$

where *w*_*ij*_ is the edge weight and ε = 10^–9^ is a small constant added inside the logarithm to avoid numerical singularities and undefined logarithms in the presence of zero or near-zero values. Additionally, the epsilon is also added outside to ensure a positive distance in case of weight of one. This transformation follows established approaches in the literature that use the negative logarithm of interaction strengths to define effective network distances.^17^

### CNR2 Plasmids, Mutagenesis, and Transfection

HEK293T cells were seeded to approximately 80% confluency in 10 cm cell culture dishes and maintained in Dulbecco’s Modified Eagle Medium (DMEM, high glucose; Thermo Fisher Scientific) supplemented with 1% fetal bovine serum and 0.1% penicillin–streptomycin (P/S). Transfection was performed using Lipofectamine 3000 Transfection Reagent (Fisher Scientific, 15212475) according to the manufacturer’s instructions. A total of 3 μg plasmid DNA per dish was used at a 2:1 ratio of receptor to pGlo-Sensor-22F (Promega), 24 h after transfection cells were seeded in Greiner 96-well white flat-bottom plates at a density of 60,000 cells per well. The plasmids used were pcDNA3.1–3 × HA-CB_2_R (cDNA Resource Center, CNR0200000) and the site-directed mutants F197^5^·^46^V (V) and G210^5^·^59^ M (M), which were synthesized and cloned into pcDNA3.1 by GeneScript (USA).

### Forskolin-Free cAMP Assay for CNR2

After 24 h of incubation, transfected HEK293T cells were equilibrated for 30 min in 80 μL per well of cAMP buffer consisting of 1× HBSS (Thermo Fisher Scientific, 14025092), 24 mM HEPES (Fisher Scientific, 10397023), 0.1% (w/v) bovine serum albumin (BSA; Sigma-Aldrich, A7906), 3.96 mM NaHCO_3_ (Fisher Scientific, 10244683), 1 mM MgSO_4_ (Fisher Scientific, 10346190), and 1.3 mM CaCl_2_·2H_2_O (Fisher Scientific, 10316313). The buffer was supplemented with 0.45 mg/mL firefly D-luciferin free acid (NanoLight Technology, 306–250) and 0.5 mM 3-isobutyl-1-methylxanthine (IBMX; Sigma-Aldrich, 15979).

Ten microlitres of the allosteric modulator EC21a (10 μM final concentration, prepared in cAMP buffer) or buffer alone (vehicle control) were added to the wells and incubated for 20 min prior to measurement. Bioluminescence was recorded using a PHERAstar FSX Plate Reader (BMG Labtech, Germany).

Five basal readings were first obtained to ensure signal stabilization. Subsequently, 10 μL of treatment ligands supplemented with 7.5 μM (final concentration) forskolin (Hello Bio, HB1348) were injected, and bioluminescence was monitored for a total of 30 cycles (1 min per cycle).

Raw luminescence values were exported from the PHERAstar FSX plate reader and analyzed using GraphPad Prism (v10.0, GraphPad Software). For each well, the luminescence signal was normalized to the mean of the initial basal readings obtained prior to ligand addition. Area under the curve was calculated and the resulting data were expressed as percentage of change to no ligand control.

Concentration–response experiments data were plotted using a nonlinear regression (log­[agonist] vs response, variable slope, Three-parameter model) to determine EC_50_ and Emax values. When applicable, the effects of the negative allosteric modulator (NAM) EC21a were quantified as the percentage change in Emax or log­(EC_50_) shift relative to the vehicle control.

### Statistics and Reproducibility

All experiments were performed in triplicate wells and repeated independently at least three times (*N* = 3). Data are presented as mean ± SD, and statistical significance between groups was determined using Welch’s *t* test.

## Results

### Effect of Dynamics on Allosteric Pocket Detection and Common Class A Microswitches

The activation mechanism for Class A receptors has been extensively studied by comparing the static protein structures in their inactive and active state.^5^ Although residue interaction networks have been widely used to study GPCR activation and signaling, the impact of extrahelical allosteric ligands on these interaction patterns remains incompletely characterized. To investigate this issue, we selected four representative systems: (1) the Complement C5A receptor (C5AR1) with the allosteric antagonist Avacopan bound, (2) the purinergic receptor (P2RY1) with the allosteric antagonist BPTU bound, (3) the Cannabinoid receptor 1 (CNR1) with the PAM ZCZ011 bound for retrospective analysis and (4) the Cannabinoid receptor 2 (CNR2) for testing the proposed protocol. We probed the impact of extrahelical allosteric modulators using Pocketron,^43^ a computational method that maps pocket formation, dynamics, and interpocket communication along extended MD trajectories to uncover transient sites and allosteric pathways.

For each allosteric ligand bound structure, we also selected and simulated an inactive structure and active state structure as reference. The progression of the extrahelical pocket volumes, averaged across three independent 1-μs simulation replicas, is reported in Figure 2. The pockets were annotated according to the GPCR class, with the involved transmembrane helices identified using the GPCRdb generic residue numbering, and, for extrahelical regions, assigning the membrane sublocation according to the procedure reported by Peter et al.^3^ (see Method: Identification and Analysis of Protein Binding Sites section).

**Figure 2:**
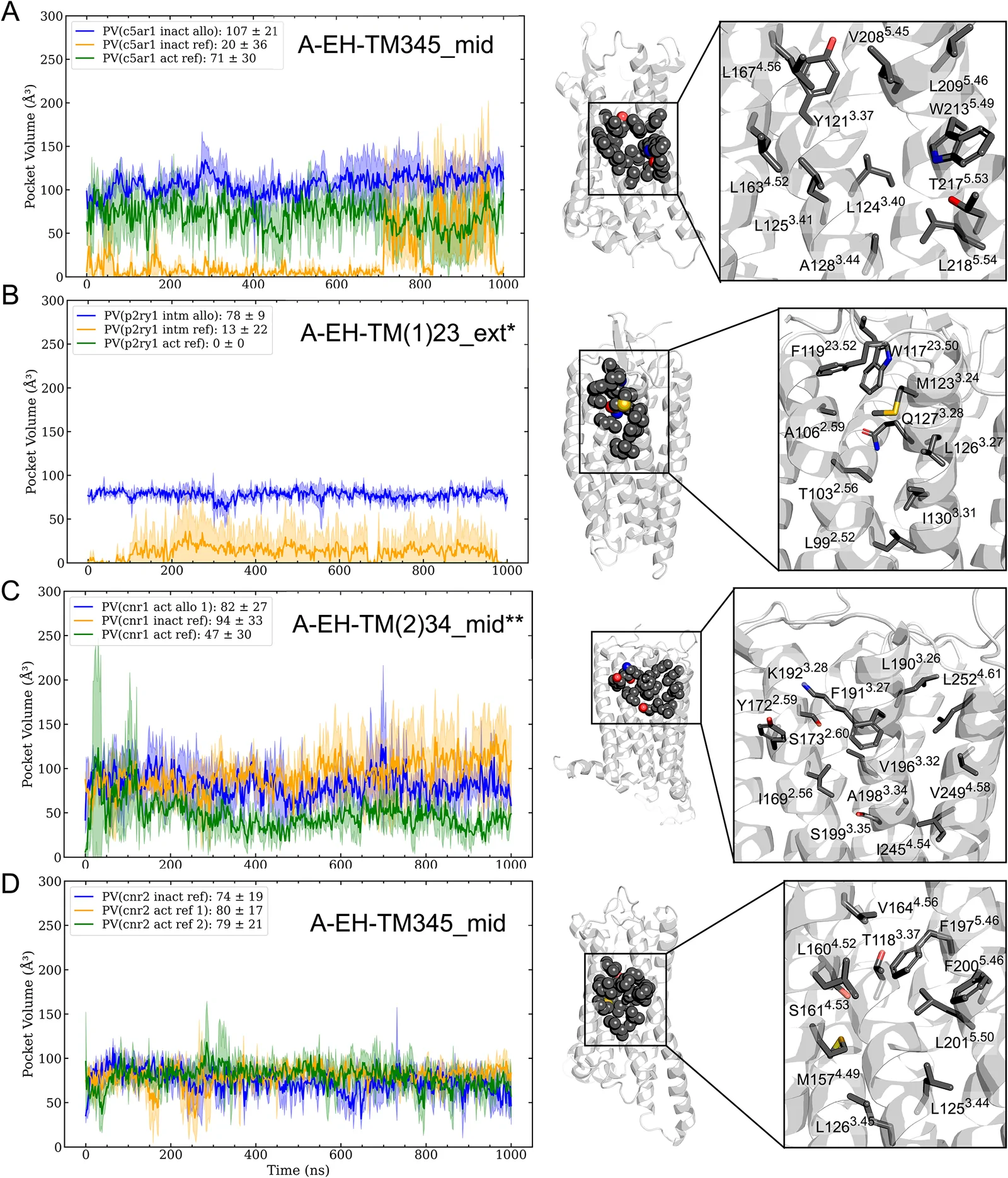
Progression of the extrahelical pocket volumes in Å^3^ for the structurally elucidated allosteric ligand-bound G protein–coupled receptor systems over 1 μs simulations, averaged across triplicates. Polar residues in each detected pocket (see Results: Effect of dynamics on allosteric pocket detection and common Class A microswitches) are highlighted. (A) Avacopan binding site in C5AR1 (A-EH-TM345_mid, gray spheres); Avacopan bound (PDB: 6C1R), unbound (orange), and active state (PDB: 7Y66, green). (B) BPTU binding site in P2RY1 (A-EH-TM123_ext, gray spheres): BPTU-bound (PDB: 4XNV, blue), inactive reference (PDB: 4XNW, orange) and active state reference (PDB: 8WJX, green). *Only subpocket A-EH-TM23_ext was detected. (C) ZCZ011 binding site in Cannabinoid Receptor 1 (CNR1) (A-EH-TM34_mid, gray spheres): ZCZ011-bound (PDB: 7WV9, blue), inactive reference (PDB: 5U09, orange) and active reference (PDB: 6KPG, green) **Interaction with TM2 occurs sporadically. (D) Mutational study^29^ suggests negative allosteric modulator (NAM) EC21a predicted to bind A-EH-TM345_mid. Pocket volume progression is shown for AM10257-bound structure (PDB: 5ZTY, blue), active state with CP55,940 (orange) and with HU308 (green) of the detected pocket (gray spheres).

We investigated the presence and volume of allosteric sites in the four case studies (Figure 2), both in the absence and presence of allosteric ligands. In parallel, we monitored the persistence of inter-residue contacts across all replicas within key structural motifs known to undergo rearrangement during GPCR activation (Figure 3A). The analyzed elements included: (i) the P^5.50^I^3.40^W^6.48^/F^6.44^ activation switch at the base of the orthosteric site, (ii) the hydrophobic L^3.43^V^6.40^V^6.41^ motif, (iii) the sodium pocket (D^2.50^, N^7.45^, S^3.39^, and N^7.49^), (iv) the N^7.49^P^7.50^xxY^7.53^ motif, and (v) the D^3.49^R^3.50^Y^5.58^ motif..

**Figure 3:**
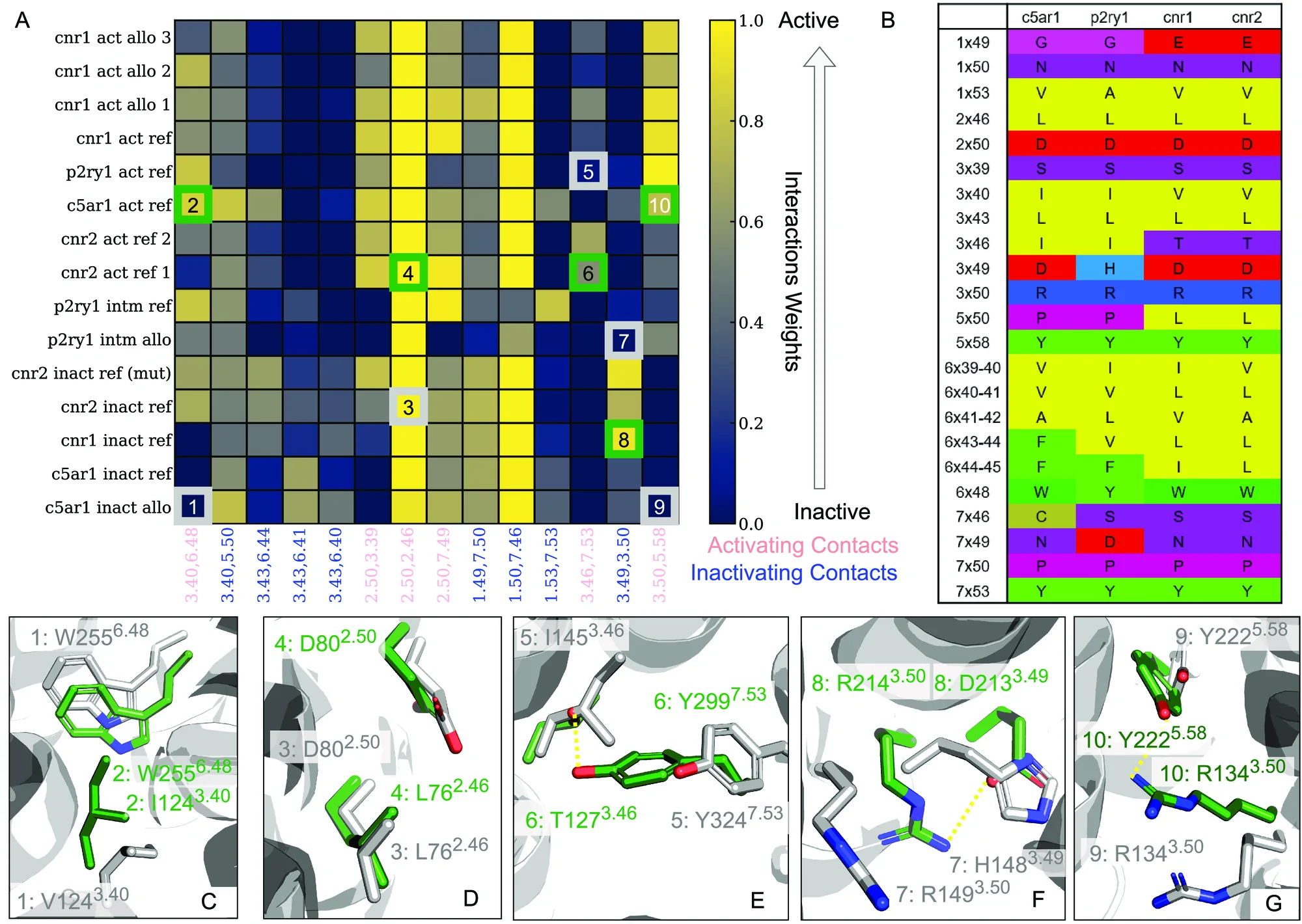
State-specific contact persistence and robustness to amino acid substitutions. (A) Heatmap showing the persistence of contacts previously implicated in GPCR activation across three 1-μs simulation replicas (Figures S1–S5). (B) Overview of amino acid residues with conserved generic numbering and coloring from GPCRdb.^30–33^ (C–G) Interacting residues shown as gray or green licorice, with hydrogen bonds depicted as yellow dashed lines. Numbering corresponds to the systems highlighted in panel (A). Abbreviations: allo, allosteric; act, active; inact, inactive; intm, intermediate; ref, reference. Systems include: C5AR1 inact allo (PDB: 6C1R), C5AR1 inact ref (PDB: 6C1R without Avacopan), CNR1 inact ref (PDB: 5U09), CNR2 inact ref (PDB: 5ZTY), CNR2 inact ref mut (PDB: 5ZTY, G2105.59M), P2RY1 intm allo (PDB: 4XNV), P2RY1 intm ref (PDB: 4XNW), CNR2 act ref 1 (PDB: 8GUR), CNR2 act ref 2 (PDB: 8GUS), C5AR1 act ref (PDB: 7Y66), P2RY1 act ref (PDB: 8WJX), CNR1 act ref (PDB: 6KPG), CNR1 act allo 1 (PDB: 7WV9), CNR1 act allo 2 (PDB: 8IKG), and CNR1 act allo 3 (PDB: 8IKH).

The first case study focused on the Avacopan binding site, A-EH-TM345_mid, at C5AR1. The binding site is also detected in both reference structures independent of the conformational state (Table 1). For C5AR1, the pocket volume is the largest if Avacopan was present during the simulation, namely 107 ± 21 Å^3^ compared to a volume of 20 ± 36 Å^3^ without Avacopan (Figure 2A). At the base of the orthosteric site, W^6.48^, which is a conserved residue functioning as an activation switch, moves closer to TM3 upon activation and forms a hydrophobic interhelical contact with residue 3.40.^46^ This rearrangement is illustrated by comparing the Avacopan-bound C5AR1 structure to the active-state reference structure (Figure 3A–C). Additionally, our simulations showed that the DRY motif exhibits activation-correlated rearrangements (Figure 3A,B,F,G).

The purinergic A-EH-TM123_ext binding site is a challenging pocket to detect as it is small and shallow.^3^ Its identification in all three replicas was only possible in the structures solved with the allosteric antagonist BPTU. The site emerged in only one of the three replicas of the inactive reference structure simulation, demonstrating ligand-induced effect of BPTU. For P2RY1, the pocket volume (74 ± 12 Å^3^) is the largest among the systems simulated in complex with the allosteric ligand (Figure 2B). This site has proven difficult to detect relying only on static structures,^3^ highlighting the key contribution of MD simulations in transiently revealing cryptic or hidden pockets. While the Avacopan binding site (A-EH-TM345_mid) could also be identified in the reference structures, indicating that static models can sometimes provide a satisfactory description, only MD simulations could account for the pronounced ligand-induced fit effects displayed by the purinergic system. Furthermore, P2RY1 uniquely features among class A GPCRs a histidine residue at position 3.49 in place of the conserved aspartic acid (Figure 3B). This substitution disrupts the stabilizing interactions that the native aspartic acid normally forms with R3.50 in the inactive state. (Figure 3F).

The final two case studies focus on CNR1 and CNR2. For CNR1, the PAM binding site, A-EH-TM34_mid, was also detected in both reference structures independent of conformational state. The PAM binding site is slightly larger, with an average volume of 82 ± 27 Å^3^, compared to the active state structure (47 ± 30 Å^3^), but smaller than the corresponding pocket in the inactive state structure, which displays an average volume of 94 ± 33 Å^3^. In 2025, Farooq et al.^29^ published mutational data supporting the idea that the negative allosteric modulator EC21a binds at the A–EH–TM345_mid site. The alanine scan identified seven residues within this pocket that were critical for allosteric signaling. Consistently, our binding site prediction identifies a corresponding pocket at A-EH-TM345_mid for CNR2, in agreement with these experimentally validated findings. In terms of volume, the predicted pocket is slightly smaller in the inactive conformation compared to the active state. We next compared the binding site residues in the putative CNR2 allosteric site, A-EH-TM345_mid, between CNR1 and CNR2 using their inactive structures (Figure 4). This comparison revealed a notable nonconserved position at 5.46, which corresponds to F197^5.46^ in CNR2 and V282^5.46^ in CNR1. We hypothesized that the presence of the bulkier phenylalanine side chain in CNR2 alters the shape and steric properties of the EC21a binding pocket relative to CNR1, thereby contributing to the distinct pharmacological profiles of EC21a observed between the two receptor subtypes. To test this hypothesis, we experimentally assessed the effect of mutating F197^5.46^ to valine in CNR2 (see Results: Experimental Insights into the Pharmacological Profiles of EC21a for CNR1 and CNR2 section).

**Figure 4:**
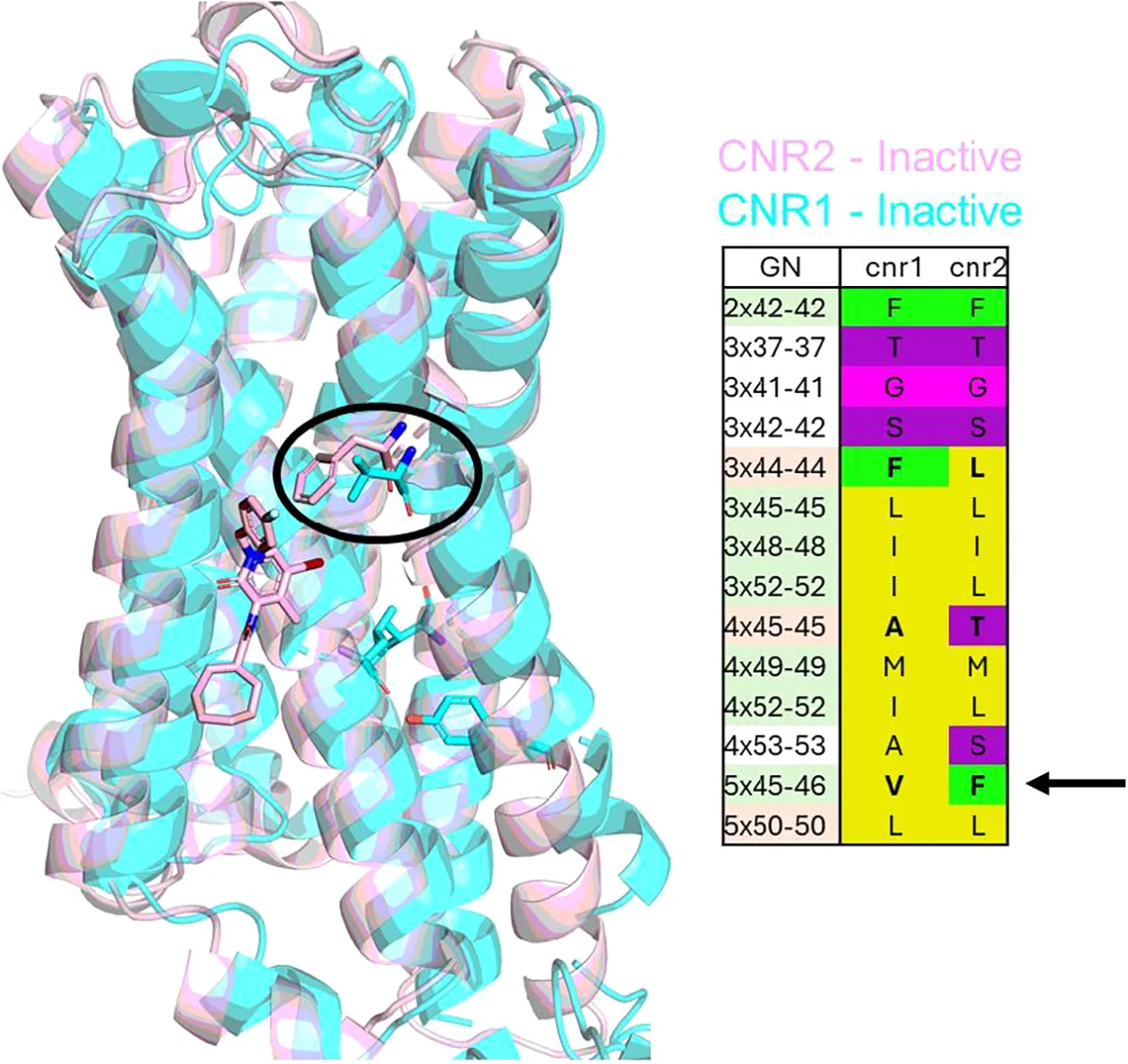
Binding-site comparison in the inactive states of Cannabinoid Receptors 1 and 2 (CNR1, cyan; CNR2, pink). Mutations that abolished or altered the allosteric signal are shown in green, whereas positions with no observed effect are shown in orange. Untested residues are shown in white. The black circle highlights the nonconserved position 5.46, F197 in CNR2 and V282 in CNR1.

Overall, our study identified most of the previously published state-specific contacts by Hauser et al.^5^ and Zhou et al.^46^ (see Table S6). These interactions were evaluated based on their persistence across three replica simulations, revealing that inactive-state contacts are enriched in inactive structures, whereas active-state contacts dominate in active conformations. Transient contacts associated with the opposing state were also detected, reflecting a dynamic fluctuation along the activation pathway. Moreover, our analysis revealed that amino acid variations and ligand-induced effects, as exemplified by P2RY1, can modulate the formation of state-specific contacts and influence the properties of the allosteric site. Interestingly, in all systems, the interaction between L^2.46^ and D^2.50^ was conserved across all systems and conformational states, whereas the interaction between R^3.50^ and Y^5.58^ clearly separates the active from inactive conformations (Figure 3A, B, D).

### Mapping of Ligand-Induced Allosteric Communication Networks (ACN)

In the investigated examples, extrahelical allosteric ligands do not simply occupy pre-existing, persistent pockets, but can alter the local interaction environment and the conformational behavior of microswitch residues in a state-dependent manner. We therefore asked whether these ligand- and state-dependent changes are also reflected in the way the orthosteric region and the intracellular interface are connected to one another. To address this question, we next analyzed the shortest communication paths linking these regions. To this end, we constructed ACNs from MD trajectories and identified the shortest paths connecting orthosteric and intracellular residues. ACNs are expressed as graphs with nodes representing residues and edges weighting the interaction between residues along MD trajectories, considering three independent replicas. We then identified the shortest path linking any orthosteric-site residue to the intracellular-site residues (see Methods: Calculation of Allosteric Communication Network section). In the graphical representation, edge thickness reflects how often the shortest paths traverse each interaction.

1. **C5AR1 activation mechanism:** In Figure 5, we report on the ACNs generated for the C5AR1 system using experimentally resolved inactive and active receptor conformations. The inactive-state ACNs were derived from the C5AR1 structure (PDB: 6C1R), analyzed both in the presence and absence of the allosteric antagonist Avacopan, whereas the active-state ACN was obtained from the agonist-activated structure (PDB: 7Y66).
  Comparison of the inactive (Figure 5B,C) and active (Figure 5D) ACNs pinpoints the rearrangement of the hydrophobic interactions involving residue M120^3.36^ as a key determinant of C5AR1 activation. In the inactive conformation, M120^3.36^ engages in a stabilizing interaction with W341^6.48^. Notably, this interaction is consistently observed both in the Avacopan-bound and ligand-free inactive structures, indicating that it represents an intrinsic inactive state-stabilizing contact rather than a ligand-dependent effect. In contrast, in the active conformation, M120^3.36^ undergoes a conformational shift and no longer interacts with W341^6.48^; instead Y290^7.42^ becomes the primary interaction partner. This reorganization of the TM3-TM6/TM7 interaction network corresponds to the activation-associated structural rearrangement of C5AR1 and is in agreement with previous structural and functional observations reported by Feng et al.^47^
2. In P2RY1, the orthosteric antagonist MRS2500 and the allosteric antagonist BPTU represent two distinct strategies for stabilizing the receptor in its inactive conformation. MRS2500 binds the orthosteric site, whereas BPTU targets the extrahelical allosteric pocket (A-EH-TM123_ext). ACN analysis of the MRS2500-bound P2RY1 structure (PDB: 4XNW) reveals the presence of allosteric communication paths connecting the orthosteric binding site to the intracellular region mediated by stabilizing interactions linking TM2, TM3, TM7 (Figure 6A). Specifically, MRS2500 promotes the interaction between Y100^2.53^ and S314^7.42^, as well as between D97^2.50^ and N134^3.35^, thereby reinforcing interhelical coupling that constrains receptor dynamics. In contrast, in the BPTU-bound structure (PDB: 4XNV), the ACN highlights a different stabilization pattern (Figure 6B). Here, the receptor inactivation is primarily achieved through reinforcement of TM2-TM7 interaction between D97^2.50^ and S317^7.46^, with fewer supporting interactions across TM3. In the active ADP-bound P2RY1 conformation (PDB: 8WJX), no shortest communication paths involving D97^2.50^, Y100^2.53^, N134^3.35^, S314^7.42^ and S317^7.46^ are observed (Figure 6C). Among these residues, Y100^2.53^ plays a critical mechanistic role by hindering the conformational rearrangement of F141^3.32^, a structural transition required for P2RY1 activation, as previously described by Li et al.^48^ Notably, while both antagonists stabilize TM2-TM7 coupling, MRS2500 enforces a more interconnected allosteric network involving TM2, TM3, and TM7, whereas BPTU relies on more localized TM2-TM7 interaction. This reduced stabilization may account for the weaker antagonistic potency of BPTU (average pEC_50_ = 6.8 ^49–53^ and p*K*_i_ = 7.6^50^) compared to MRS2500, which exhibits substantially higher affinity and efficacy (average p*K*_i_ = 9.1 ^54^ and pEC50= 8.0 ^49,55^ ).
3. **CNR1 biased agonism:** For the CNR1 systems, we investigated the sensitivity of our ACN approach to detect ligand-induced signaling bias. In 2024, Shen et al.^56^ reported that demethylation of the nonbiased PAM ZCZ011 converts the scaffold into Gi-biased allosteric agonists, reducing adverse effects such as addiction, hypothermia, and catalepsy, while maintaining its desirable properties in pain management. Their central hypothesis is that the biased ligand displays a less tight interaction with TM2.
  Consistent with this hypothesis, our ACN analysis of the ZCZ011-bound CNR1 structure (PDB: 7WV9) shows a well-defined shortest communication path traversing TM2, involving coupling between S158^2.45^ (TM2) and S206^3.42^ (TM3), as well as stabilizing interactions with key binding-site residues including I169^2.56^ (Figure 7A). In contrast, for the Gi-biased ligand CB-02 (PDB: 8IKG), loss of the methyl interaction with I169^2.56^ weakens TM2-centered communication, reflected by a disrupted TM2-TM4 interaction between S158^2.45^ and W241^4.50^ (Figure 7B).
  This effect is further amplified for the more potent Gi-biased agonist CB-05 (PDB: 8IKH), where the shortest allosteric communication path is completely rerouted and no longer propagates through TM2 or its associated TM2-TM3-TM4 interaction network (Figure 7C). Altogether, these results demonstrate that progressive loss of TM2-mediated allosteric communication correlates with Gi-biased signaling, in agreement with experimental observations reported by Shen et al.^56^
4. **CNR1 and CNR2 allosteric signaling:** Finally, we were interested in investigating differences between CNR1 and CNR2 ACNs. Farooq et al.^29^ published a study combining computational analysis and alanine scanning, supporting the presence of a CNR2 allosteric site at A-EH-TM345_mid. This is considered the putative binding site of allosteric modulator EC21a, which, interestingly, displays a different pharmacological profile for CNR1 and CNR2. EC21a works: (i) as PAM-antagonist for CNR1, blocking signaling but increasing the affinity for the agonist and (ii) as NAM in combination with the orthosteric ligands JWH133 in CNR2. Because the binding pose of EC21a has not been experimentally established, we did not perform simulations of receptor systems explicitly including EC21a. Comparing the ACNs of CNR1 and CNR2, focusing on the cross-talk between A-EH-TM345_mid and the orthosteric site, we observed that in the antagonist-bound inactive conformation of CNR2 (AM10257-bound structure, PDB: 5ZTY), the TM5-TM6 interface is stabilized by the shortest communication path linking Y209^5.58^ and G248^6.38^ (Figure 8A). This interaction forms a continuous allosteric communication network across the transmembrane core. In contrast, in the taranabant-bound inactive CNR1 structure (PDB: 5U09), no corresponding TM5-TM6 allosteric pathway is detected between the conserved residues Y294^5.58^ and G346^6.38^, indicating more flexibility between these helices in this receptor subtype (Figure 8C).

**Figure 5:**
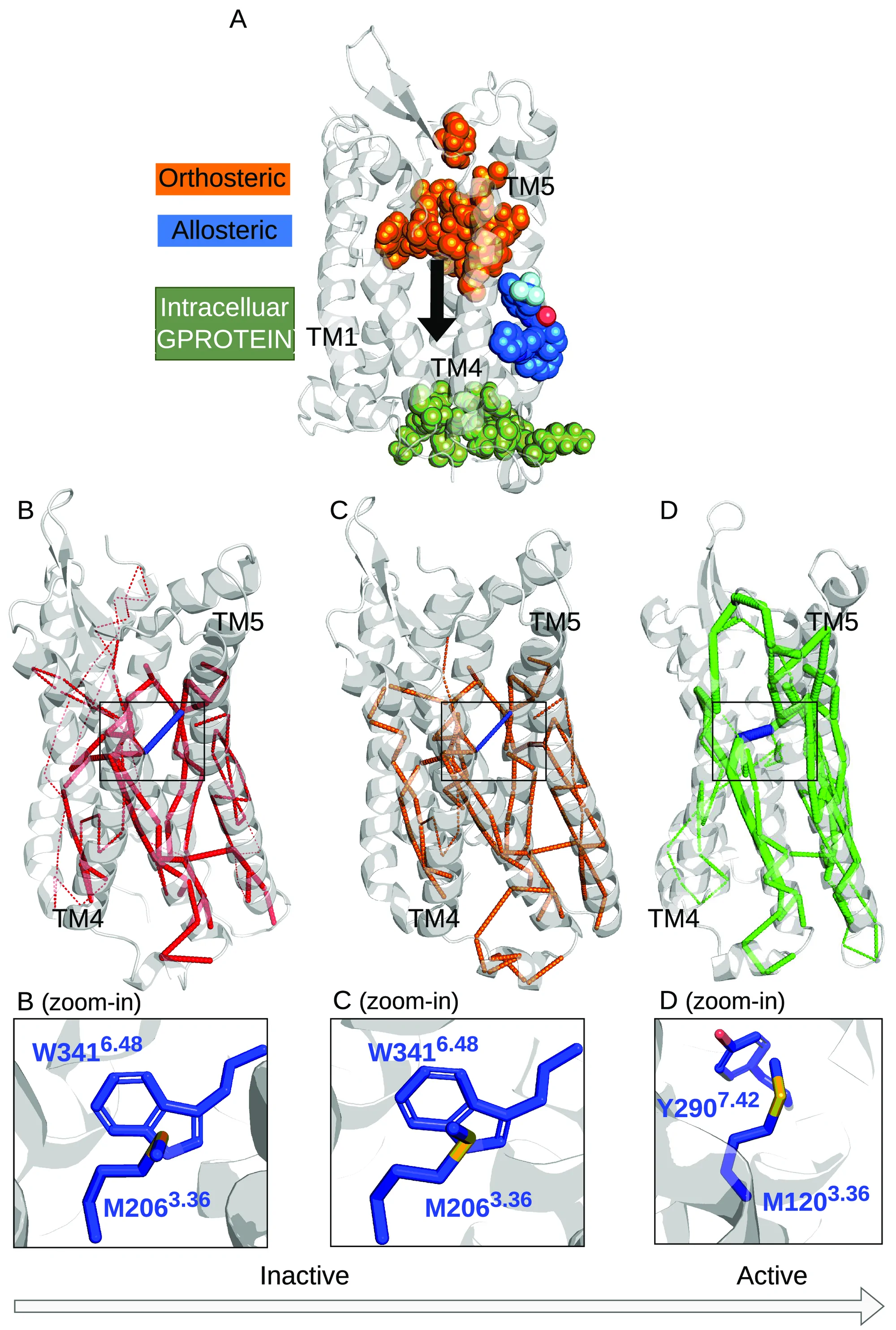
Allosteric Communication Pathway (ACN) for the Complement C5a receptor (C5AR1). (A) Gray cartoon representation of the C5AR1 receptor (PDB: 6C1R) highlighting the orange orthosteric residues, the green intracellular residues and the allosteric antagonist Avacopan in blue. (B) ACN of the Avacopan bound C5AR1 (PDB: 6C1R) in gray cartoon connecting Cα atoms based on shortest-path distance evaluation between the orthosteric and intracellular site. Nodes represent amino acid residues and the edge of the interaction between two amino acid residues. The thicker the edge, the more often the edge was part of the shortest path connecting the orthosteric with the intracellular site. The interaction between W341^6.48^ and M206^3.36^ is highlighted in blue. (C) Illustration of the ACN for C5AR1 (PDB: 6C1R) in gray cartoon without Avacopan. Similarly to the Avacopan bound structure, the inactive state stabilizing interaction between residues W341^6.48^ and M206^3.36^ is highlighted in blue. (D) ACN for C5AR1 receptor (PDB: 7Y66) in the active state, where M120^3.36^ shifts instead of W189^6.48^ interacts with Y290^7.42^ as previously reported by Feng et al.^47^

**Figure 6:**
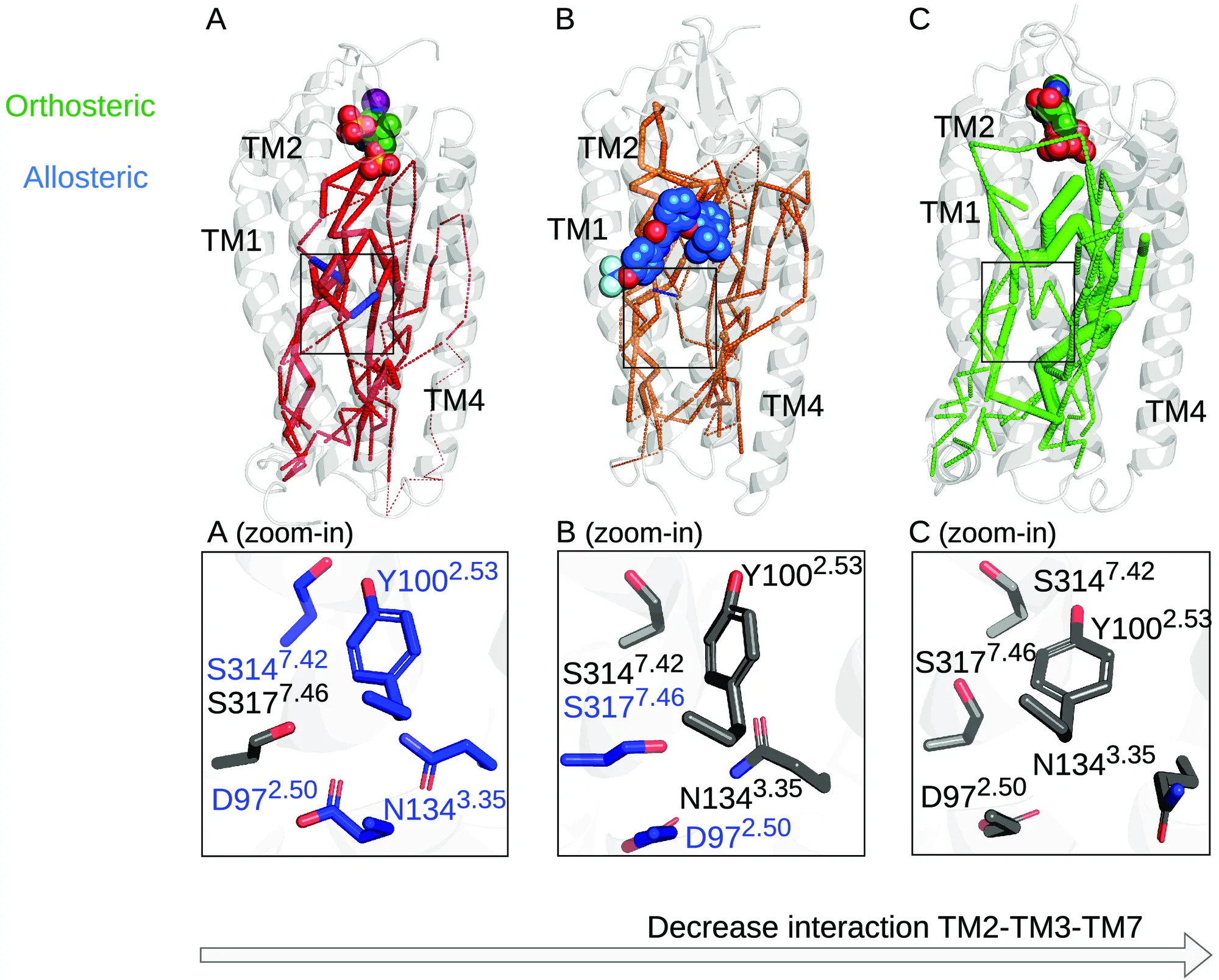
Allosteric Communication Pathway (ACN) for the Purinergic Receptor P2Y (P2RY1). (A) Paths connecting the orthosteric site to the intracellular site for P2RY1 receptor bound to the antagonist MRS2500 (PDB: 4XNW) in green spheres. MRS2500 stabilizes the interactions between Y100^2.53^ and S314^7.42^ and D97^2.50^ and N134^3.35^. (B) Representation of the P2RY1 receptor in gray cartoon with the allosteric antagonist BPTU in blue spheres (PDB: 4XNV). The effect of BPTU in stabilizing the TM2-TM7 interactions between D97^2.50^ and S317^7.46^. (C) The ACN network for the ADP-bound active P2RY1 conformation (PDB: 8WJX) does not show any shortest path interactions between residues D97^2.50^, Y100^2.53^, N134^3.35^, S314^7.42^ and S317^7.46^.

**Figure 7:**
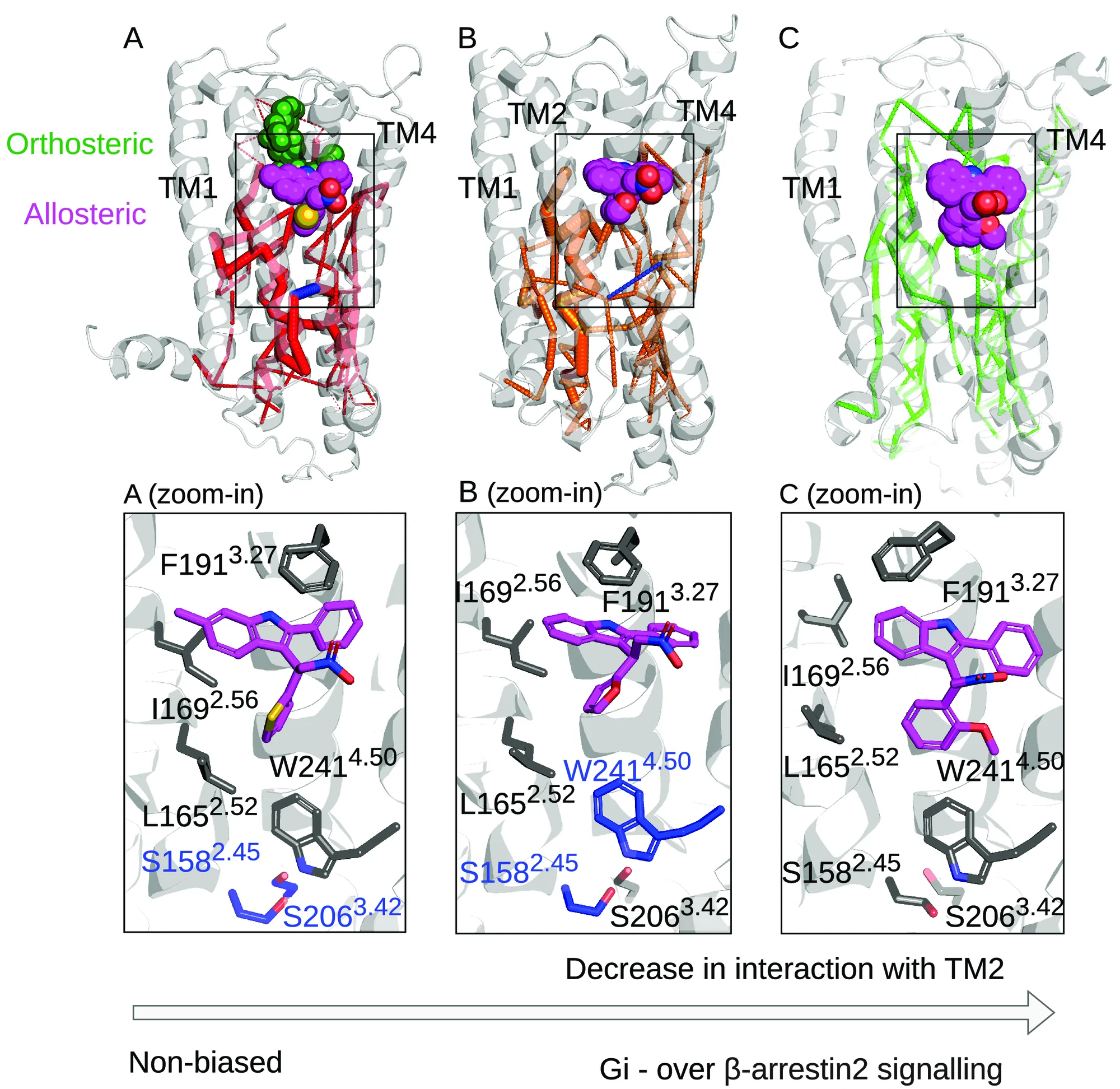
Investigating biased signaling of Cannabinoid Receptor 1 (CNR1). (A) Allosteric Communication Network (ACN) of the nonbiased PAM ZCZ011-bound (magenta spheres) and agonist CP55940-bound (green spheres) CNR1 structure (PDB: 7WV9). Key binding site residues, including F191^3.27^, I169^2.56^, and L165^2.52^, are depicted as gray licorice. The TM2-TM3 interaction between S158^2.45^ and S206^3.42^ is highlighted in blue. (B) ACN of G_i_-biased allosteric agonist CB-02 (PDB: 8IKG) illustrates that the loss of the methyl interaction with I2.56 results in a weaker interaction between TM2-TM4 residues S158^2.45^ and W241^4.50^. (C) The ACN of the more potent G_i_-biased allosteric agonist CB-05 (PDB: 8IKH) in magenta spheres shows that the shortest path indicated as green interaction does not lead anymore through S158^2.45^ with either S206^3.42^ or W241^4.50^ demonstrating that TM2-TM3-TM4 interaction loss induces Gi-biased signaling as previously reported by Shen et al.^56^

**Figure 8:**
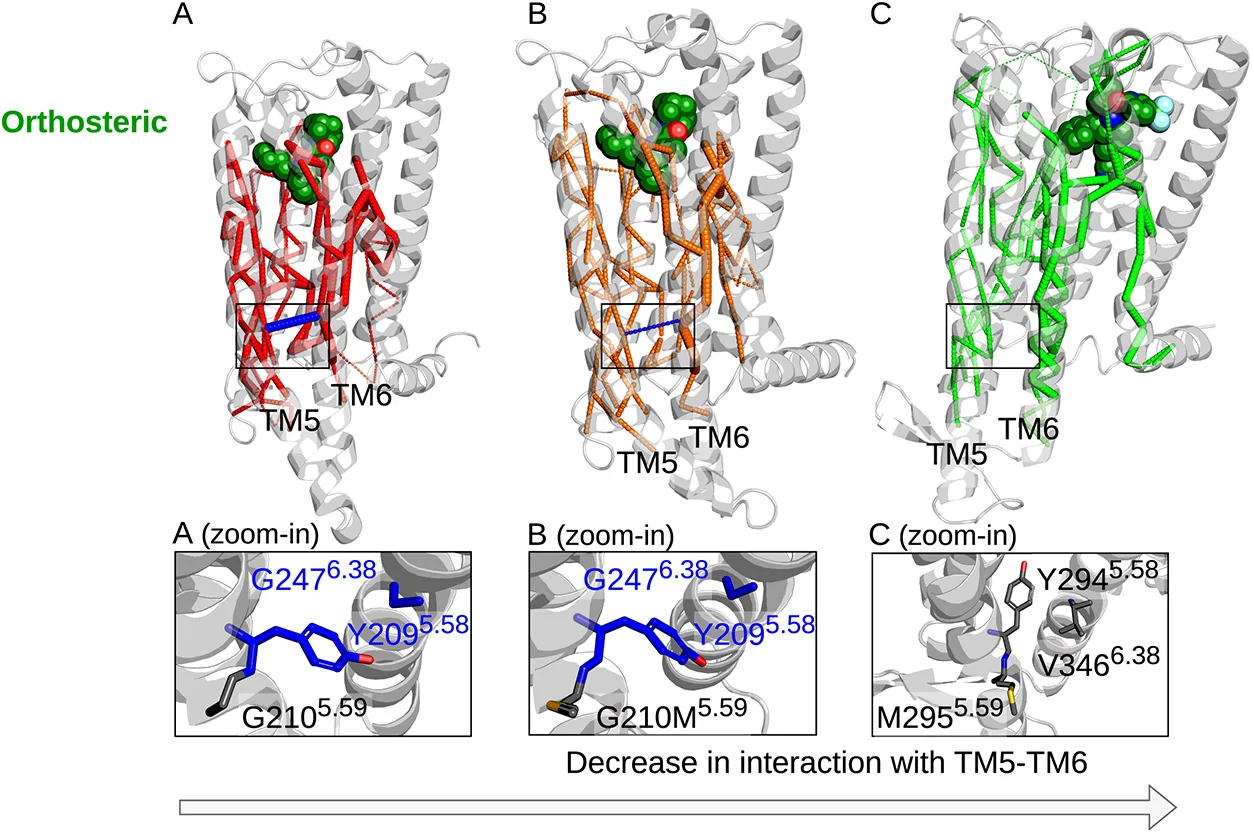
Allosteric Communication Pathway (ACN) in the inactive conformation of the Cannabinoid Receptor 1 and 2 (CNR1/CNR2). (A) Illustration of the ACN represented in red of the antagonist AM10257 (green spheres)-bound CNR2 structure (PDB: 5ZTY) in gray cartoon. TM5-TM6 are connected via the residues Y209^5.58^ and G248^6.38^. (B) Simulation of the antagonist AM10257-bound structure with a mutation of G210M^5.59^ weakens the interaction between Y209^5.58^ and G248^6.38^ as shown in blue. (C) For the taranabant-bound CNR1 structure (PDB: 5U09) depicted as green spheres, the ACN is not linked via Y294^5.58^ and G346^6.38^ indicating that the stabilization of the link could be a selectivity driver between CNR1 and CNR2.

A detailed residue-level analysis revealed that, adjacent to Y209^5.58^, CNR2 carries a glycine at position 5.59, whereas CNR1 contains a bulkier methionine at the same position. Consistently introducing a G210^5.59^ M mutation in CNR2 weakens the Y209^5.58^–G248^6.38^ interaction and disrupts the TM5-TM6 allosteric communication network (Figure 8B), phenocopying the CNR1-like behavior. These results suggest that subtle differences in the TM5 sequence can modulate long-range allosteric signaling and may contribute to the subtype-specific negative allosteric modulation observed for EC21a in CNR2.

### Experimental Insights into the Role of A-EH-TM345_mid in EC21a Pharmacology at CNR1 and CNR2

To investigate whether the differential pharmacological effects of EC21a at CNR1 and CNR2 could be associated with subtype-dependent differences, either static or dynamic, in the A-EH-TM345_mid site, we tested two mutations. F197^5.46^V was chosen to probe a nonconserved residue within the reported EC21a site, whereas G210^5.59^ M was chosen to test a position implicated by the ACN analysis in allosteric communication. For F197^5.46^V, the modest decrease in ΔpEC_50_, from 1.06 ± 0.30 (WT) to 0.93 ± 0.41 was within experimental error and therefore does not support a major contribution of F197^5.46^ to the observed pharmacological effect under the conditions tested. However, a more subtle or context-dependent role of this residue in shaping the local properties of the putative A-EH-TM345_mid site cannot be excluded (Table 2 and Figure S6). In contrast, the second mutation, G210^5.59^ M, strongly altered the EC21a NAM response, with ΔpEC_50_ increasing by more than 5σ, from 1.06 ± 0.30 (WT) to 2.49 ± 0.19 (Figure S6E,F). This result supports the functional relevance of position 5.59 for allosteric modulation, although the direction of the effect indicates a more complex relationship between local network features and global pharmacological outcome than initially anticipated. The role of G210^5.59^ was previously described by Hua et al.^57^ as providing flexibility within TM5. The G210^5.59^ M mutation increases the side-chain volume approximately 3-fold, introducing steric bulk that likely stabilizes TM5 in its inactive conformation through steric hindrance. In addition, Cordomí et al.^58^ reported an aromatic interaction between residues at positions 5.59 and 5.63. The methionine substitution introduces a side chain that may engage in favorable interactions with the tryptophan at position 5.63, further contributing to the stabilization of TM5.

**Table 2:** Summary of the Mean (*n* = 3) pEC_50_ and ΔpEC_50_ for FK-Induced cAMP Release in CNR2 WT, F197^5.46^V, and G210^5.59^ M^a^

	pEC_50_	ΔpEC_50_
CNR2 WT	8.10 ± 0.22	1.06 ± 0.3
CNR2 WT+ EC21a	7.03 ± 0.48*	
CNR2 V	7.13 ± 0.68	0.93 ± 0.41
CNR2 V + EC21a	6.20 ± 0.23	
CNR2M	8.59 ± 0.13	2.49 ± 0.19*
CNR2M + EC21a	6.09 ± 0.32**	

a HEK293T cells were treated with the agonist JWH133 and in the presence or absence of EC21a. The pEC_50_ values were derived from concentration–response curves and compared to JWH133 alone. The ΔpEC_50_ values were compared to CNR2 WT. *P < 0.05; **P < 0.01.

## Discussion

Targeting GPCRs through the rational design of allosteric modulators opens new avenues for drug discovery by standardizing GPCR allosteric communication networks and enabling a more robust comparison between results from different receptors. However, to bridge the current knowledge gap regarding which allosteric sites are functionally relevant, a deeper understanding of how allosteric ligands influence the dynamic communication between the orthosteric site and the G protein binding interface is essential. To this end, we performed 45 μs of classical molecular dynamics (MD) simulations across four pharmaceutically relevant systems that differ in their endogenous binding partners. Our analysis shows that calculating ACNs between orthosteric and intracellular residues can reveal rearrangement in key structural motifs and highlight the features of biased agonism. Our results reveal interactions that, aside from the interaction between Y209^5.58^ and G248^6.38^, have not been described by Hauser et al.^5^ or Zhou et al.^46^ This approach is likely transferable across all Class A GPCRs. For example, it could be applied to the recently published comprehensive MD data set by Aranda-García et al.,^59^ as both the orthosteric and G-protein binding sites are well-defined for class A GPCRs, eliminating the need to decide which ACN interactions to postfilter.

Despite these insights, several limitations must be acknowledged. First, the predictive power of ACN analysis is inherently constrained by the thoroughness of MD sampling. In line with best practices, we mitigated sampling limitations by independently running the simulations three times. Furthermore, our analysis focused on Cartesian coordinate displacements, thereby only implicitly capturing potentially informative metrics such as dihedral angles, electrostatic interactions, and water-mediated contacts. These simplifications may limit the resolution of our predictions, particularly for global signaling effects. In this regard, the case of the predicted binding site for EC21a illustrates the complexity of allosteric modulation. Our ACN analysis successfully identified key residues that play a central role in shaping the allosteric communication network. However, while these results provide valuable mechanistic insight, predicting the global functional consequences of perturbing these residues remains a challenge. A representative limitation is illustrated by the G210^5.59^ M mutation. This substitution was initially motivated by the observation that CNR1 carries a methionine at the equivalent position and is less susceptible to EC21a than CNR2. However, introducing methionine at position 5.59 in CNR2 did not produce a simple CNR1-like pharmacological profile. Instead, it increased susceptibility to EC21a. This result indicates that, although the ACN analysis identified position 5.59 as functionally relevant, the global pharmacological consequences of perturbing this site cannot be inferred from local network features alone. Future studies integrating complementary computational and experimental approaches will be essential to translate ACN-based findings into quantitative predictions of receptor activity. To this aim, although conventional molecular dynamics simulations coupled with network analysis provide valuable insight, the inherent complexity and large size of these systems call for more sophisticated approaches.^14^ In particular, adopting enhanced sampling techniques represents a promising strategy to achieve a more detailed and accurate understanding of ACNs.

## Conclusion

Overall, this work advances allosteric GPCR research by demonstrating that dynamic molecular behavior shapes receptor function and allosteric modulation. We highlight the importance of incorporating receptor dynamics to understand the behavior of Class A microswitches through extensive molecular dynamics simulations and allosteric communication networks (ACN). In line with previous network-based studies of protein dynamics, interactions were quantified here based on their persistence over the simulation trajectory rather than by a binary presence/absence criterion derived from static structures. We applied this approach to four case studies. In C5AR1, we characterized the activation switch involving W6.48 and its communication with TM3, offering mechanistic insight into receptor activation. In P2RY1, we showed that the allosteric antagonist BPTU and orthosteric antagonist MRS2500 differently stabilize the TM2-TM7 interaction. This behavior is not observed in the ADP-bound active state, revealing how different ligands shape intracellular signaling pathways in distinct ways. We also uncovered features of biased signaling in CNR1, where residue interaction network alterations provide a structural basis for ligand-specific signaling preferences. Finally, our comparative analysis of the A-EH-TM345_mid binding site in CNR1 and CNR2 highlighted G210^5.59^ as a residue of functional relevance to subtype-dependent allosteric modulation. Collectively, these findings illustrate the utility of ACN analysis for interpreting GPCR signaling and generating experimentally testable hypotheses on allosteric effects and subtype selectivity. They further support the view that receptor conformational dynamics play a central role in allosteric regulation and signaling bias, with potential implications for the rational development of next-generation GPCR therapeutics.

## Supplementary Material





## Data Availability

The AlloViz weighted interaction data for the three replicates of the Complement C5a Receptor (C5AR1) bound to Avacopan and PMX53 peptide and of the Purinergic Receptor P2Y (P2RY1) bound to BPTU are provided in the Supporting Data Excel file. The source data is freely available in Zenodo: 10.5281/zenodo.21721558.

## Notes

The total pocket count for the three MD replicates of the Complement C5a receptor (C5AR1), the Purinergic Receptor P2Y (P2RY1), and the Cannabinoid Receptor 1 (CNR1). Table S6 provides an overview of the published 51 state-specific interactions captured by Hauser et al.^5^ and Zhou et al.^46^ and the average interaction from 0 to 1 over the 1 μs (*N* = 3) MD simulation in brackets (Tables S1–S5); the Root Mean Square Deviation (RMSD) of the Cα backbone for all systems discussed as part of this manuscript. The allosteric communication network was calculated as the mean of the three replicates (Figures S1–S5); Figure S6 shows the effect of F197^5.46^V and G210^5.59^ M mutation on FK-induced cAMP release results for Cannabinoid Receptor 2 (CNR2) (PDF)
Source Data of AlloViz calculation for the three replicates of the Complement C5a Receptor (C5AR1) bound to Avacopan and PMX53 peptide and of the Purinergic Receptor P2Y (P2RY1) bound to BPTU (XLSX)

## Abbreviations

ACN: Allosteric Communication Networks
ADRB2: β2-adrenergic receptor
C5AR1: complement C5a receptor
CNR1/CNR2: cannabinoid receptor 1 and 2
EH: extrahelical
GPCRs: G-protein-coupled receptors
ICL: intracellular Loop
MD: molecular dynamics
NAM: negative allosteric modulator
OPM: orientations of proteins in membranes
P2RY1: purinergic receptor P2Y
PDB: Protein Data Bank
POPC: 1-palmitoyl-2-oleoly phosphatidylcholine
TM: transmembrane helix
