# Supplementary material for: Residue Interaction Network Reveals Allosteric Pathways Linking Orthosteric and Intracellular Sites in Class A GPCRs
Source: J Chem Inf Model. 2026 Aug 20;66(17):10956–70. doi: 10.1021/acs.jcim.6c00526 (PMC13580027; doi:10.1021/acs.jcim.6c00526)
Supplement: Supplementary file 1 [file ci6c00526_si_001.pdf]

Supporting Information: Residue Interaction Network Reveals Allosteric Pathways Linking Orthosteric and Intracellular Sites in Class A GPCRs

*Sonja Peter[1,2], Georges Chalhoub[3], Peter J. McCormick[3], Chris De Graaf[4], Ijen Chen[1], Giovanni Bottegoni[2,5]**

[1]Nxera Pharma UK, Cambridge, CB21 6DG, UK

[2]Department of Biomolecular Sciences, University of Urbino Carlo Bo, Urbino, 61029, Italy

[3]Institute of Systems, Molecular & Integrative Biology, University of Liverpool, Liverpool, L69 7ZX, UK

[4]Structure Therapeutics, San Francisco, 94080, USA

[5]Department of Pharmacy, University of Birmingham, Birmingham, B15 2TT, UK,

giovanni.bottegoni@uniurb.it

Brief description:

- Table S1 – S5 provide information of the total pocket count for the three MD replicates of the Complement C5a receptor (C5AR1), the Purinergic Receptor P2Y (P2YR1), and the Cannabinoid Receptor 1 (CNR1). Table S6 provides an overview of the published 51 state-specific interactions captured by Hauser et al.[5] and Zhou et al.[46] and the average interaction from 0 to 1 over the 1 μs (N = 3) MD simulation in brackets.
- Figure S1 – S5 displays the Root Mean Square Deviation (RMSD) of the Cα backbone for all systems discussed as part of this manuscript. The allosteric communication network was calculated as the mean of the three replicates. Figure S6 shows the effect of F197$^{5.46}$V and G210$^{5.59}$M mutation on FK-induced cAMP release results for Cannabinoid Receptor 2 (CNR2).

Table S1. Total pocket count over 0, 200, 400, 600, 800, and 1000 ns for Complement C5a receptor (C5AR1) (PDB: 6C1R) bound by Avacopan.

Duration [ns]	Pockets [#]
0	22
100	74
200	79
400	84
600	88
800	97
1000	98

Table S2. Total pocket count over 0, 200, 400, 600, 800, and 1000ns for Complement C5a receptor (C5AR1) (PDB: 6C1R) bound without Avacopan.

Duration [ns]	Pockets [#]
0	20
100	71
200	76
400	81
600	84
800	90
1000	93

Table S3. Total pocket count for 1000ns for three replicates of Complement C5a receptor (C5AR1) (PDB: 6C1R) with and without Avacopan bound.

Systems	Pockets [#] after 1000 ns
C5AR1_6C1R_EFD	98
C5AR1_6C1R_EFD_1	97
C5AR1_6C1R_EFD_2	100
C5AR1_6C1R_apo	93
C5AR1_6C1R_apo_1	94
C5AR1_6C1R_apo_2	94

Table S4. Total pocket count for 1000ns for three replicates of the Purinergic Receptor P2Y (P2YR1) (PDB: 4XNV and 4XNW) with and without the allosteric antagonist BPTU bound.

Systems	Pockets [#] after 1000 ns
P2RY1_4XNV_BUR	120
P2RY1_4XNV_BUR _1	106
P2RY1_4XNV_BUR _2	110
P2RY1_4XNV_2ID	92
P2RY1_4XNV_2ID _1	99
P2RY1_4XNV_2ID _2	104

Table S5. Total pocket count for 1000ns for three replicates of Cannabinoid Receptor 1 (CNR1) (PDB: 7WV9 and 6KPG) with and without the positive allosteric ligand ZCZ011 bound.

Systems	Pockets [#] after 1000 ns
CNR1_7WV9_9GF7IC	114
CNR1_7WV9_9GF7IC _1	104
CNR1_7WV9_9GF7IC _2	115
CNR1_6KPG_8D0	110
CNR1_6KPG_8D0_1	111
CNR1_6KPG_8D0_2	111

Table S6. Overview the previously published 51 state-specific interactions captured by Alloviz by Hauser et al.[5] and Zhou et al.[46] and the average interaction from 0 to 1 over the 1 μs (N = 3) MD simulation in brackets. In the 13 systems which were either in the Active or Inactive conformation, on average the state-specific matched the conformation. For the intermediate structures in the case of P2RY1 (PDB: 4XNV), on average the inactive state specific contacts were more present, while for P2RY1 PDB: 4XNW, the active state-specific contacts were more present. Abbreviations of the protein families: Complement C5a receptor (C5AR1), the Purinergic Receptor P2Y (P2YR1), and the Cannabinoid Receptor 1 and 2 (CNR1/CNR2).

Systems	Conformation	Active (22)	Inactive (29)
C5AR1_6C1R_EFD	Inactive	10 (0.513)	**22 (0.553)**
C5AR1_6C1R_apo	Inactive	9 (0.525)	**22 (0.533)**
C5AR1_7Y66_apo	Active	**17 (0.533)**	18 (0.462)
CNR1_5UO9_7DY	Inactive	12 (0.451)	**22 (0.586)**
CNR1_6KPG_8D0	Active	**21 (0.669)**	13 (0.511)
CNR1_7WV9_9GF7IC	Active	**21 (0.651)**	19 (0.395)
CNR1_8IKG_Q2B	Active	**21 (0.570)**	17 (0.356)
CNR1_8IKH_Q2L	Active	**21 (0.565)**	17 (0.389)
CNR2_5ZTY_9JU_A_G210M	Inactive	**15 (0.397)**	22 (0.647)
CNR2_5ZTY_9JU_A	Inactive	17 (0.392)	**22 (0.600)**
CNR2_8GUR_9GF_AR	Active	**21 (0.607)**	14 (0.531)
CNR2_8GUS_KO3_AR	Active	**21 (0.631)**	19 (0.380)
P2RY1_4XNV_BUR_A	Intermediate	15 (0.319)	19 (0.406)
P2RY1_4XNW_2ID_A	Intermediate	16 (0.454)	23 (0.446)
P2RY1_8WJX_ADP_AR	Active	**17 (0.613)**	17 (0.442)

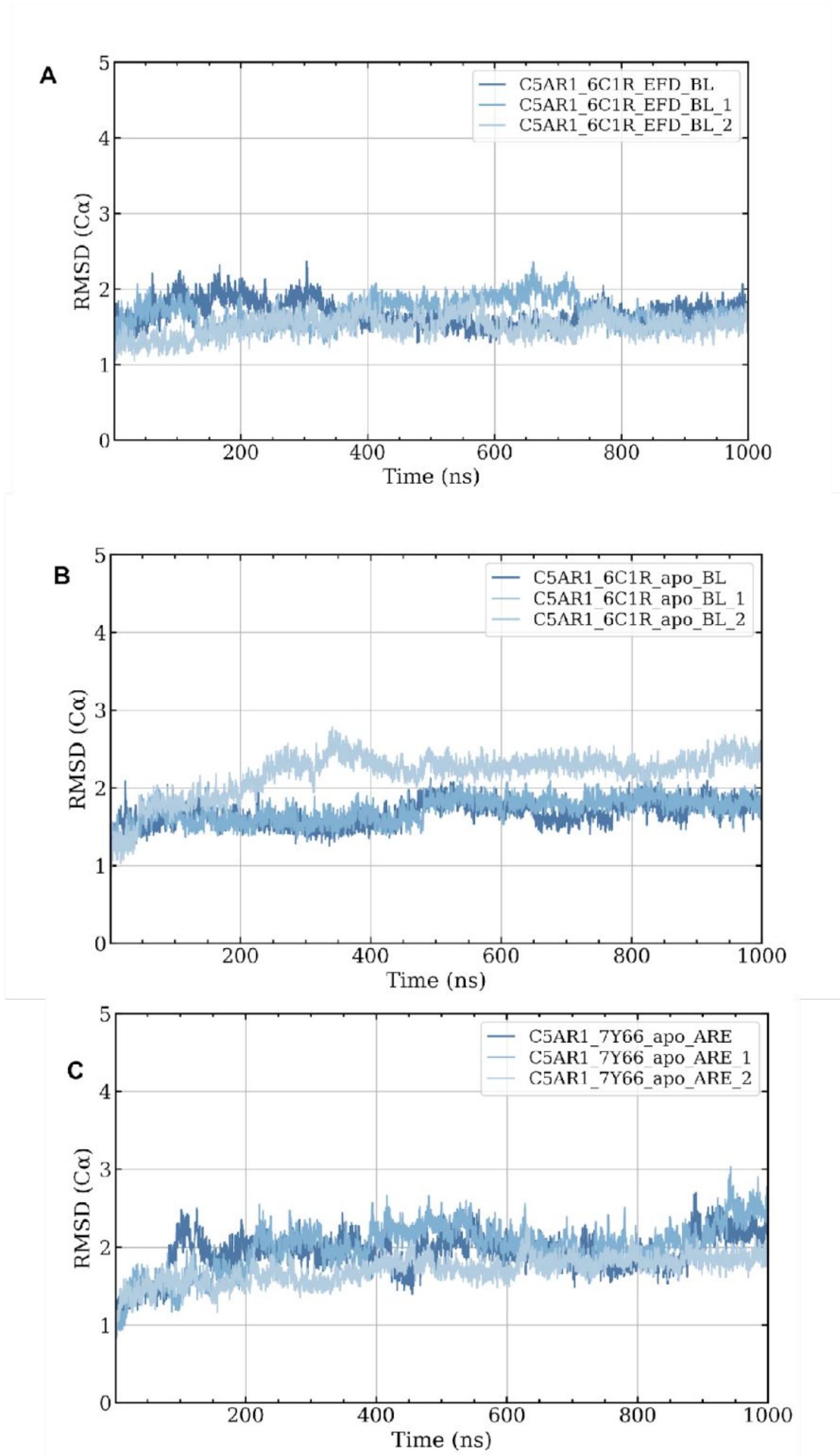

Figure S1. Root Mean Square Deviation (RMSD) of the Cα backbone for three Complement C5a receptor (C5AR1) systems over 1 μs with three replicas each. (A) The trajectories of Avacopan-bound (A) and without the Avacopan-bound structure (PDB: 6C1R), (C) the trajectories of the reference in the inactive conformation (PDB: 7Y66).

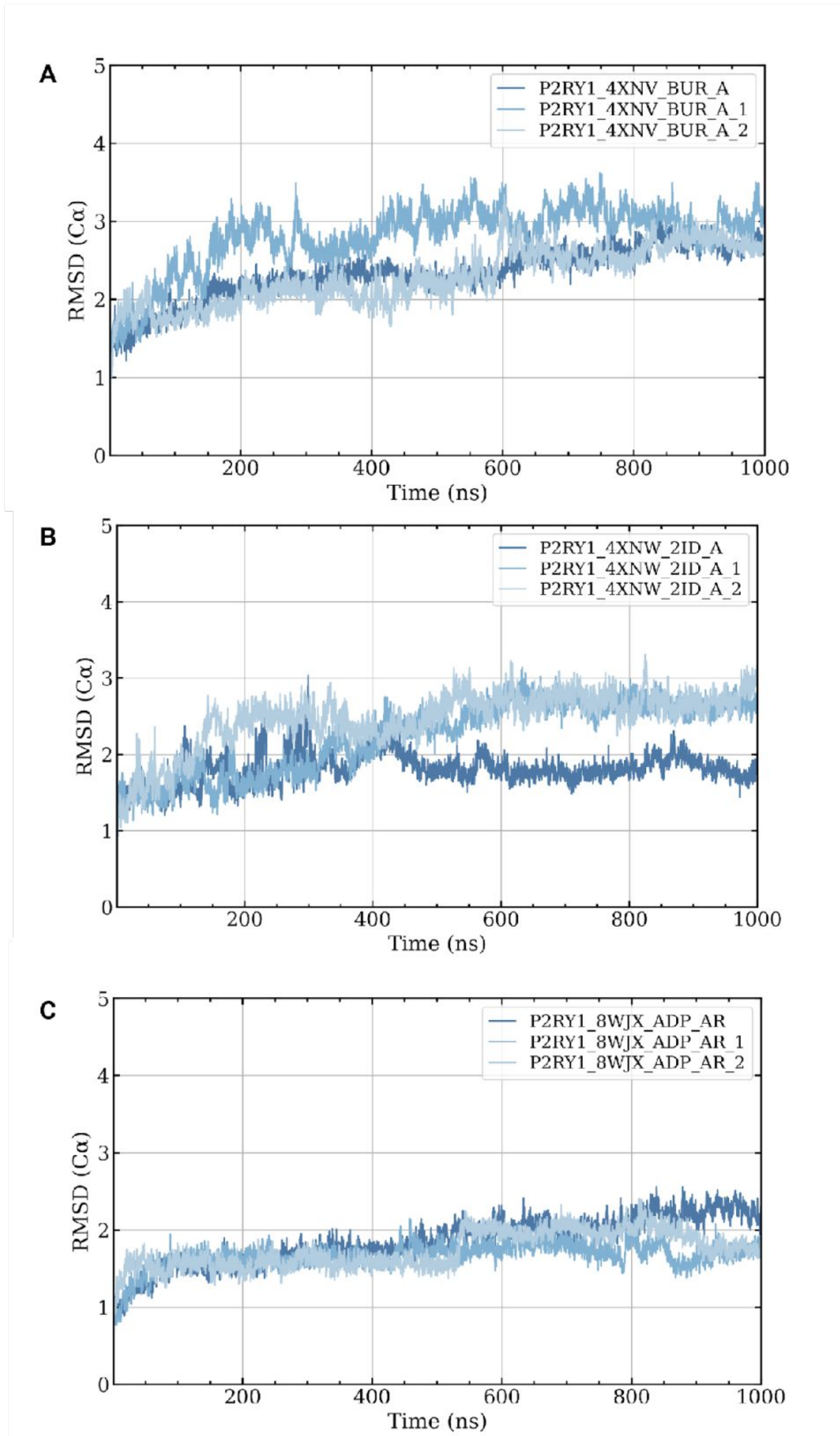

Figure S2. Root Mean Square Deviation (RMSD) of the Cα backbone for three Purinergic Receptor P2Y (P2YR1) over 1 μs with three replicas each. (A) The trajectories of the allosteric antagonist BPTU-bound structure (PDB: 4XNV), (B) the simulation of the reference in the inactive conformation bound with the

antagonist MRS2500-bound structure (PDB: 4XNV), (C) the trajectories of reference in the active conformation bound to the agonist 2ADP (PDB: 8WJX).

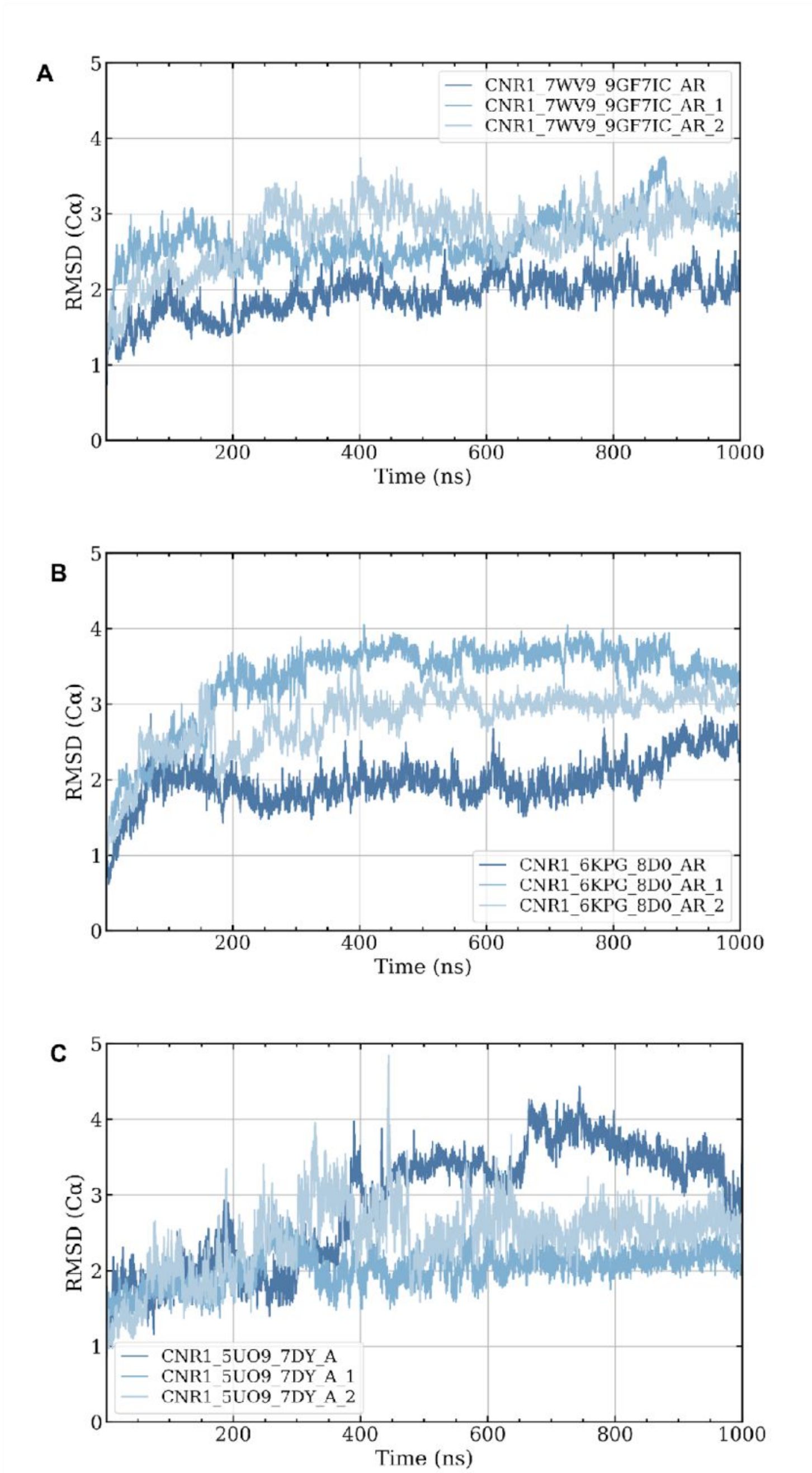

Figure S3. Root Mean Square Deviation (RMSD) of the Cα backbone for three Cannabinoid 1 Receptor (CNR1) systems over 1 μs with three replicas each. (A) The trajectories of the PAM ZCZ011-bound structure (PDB: 7WV9), (B) the simulation of the reference in the active conformation bound with the

agonist AM841-bound structure (PDB: 6KPG), (C) the trajectories of the reference in the inactive conformation bound by the antagonist taranabant (PDB: 5U09).

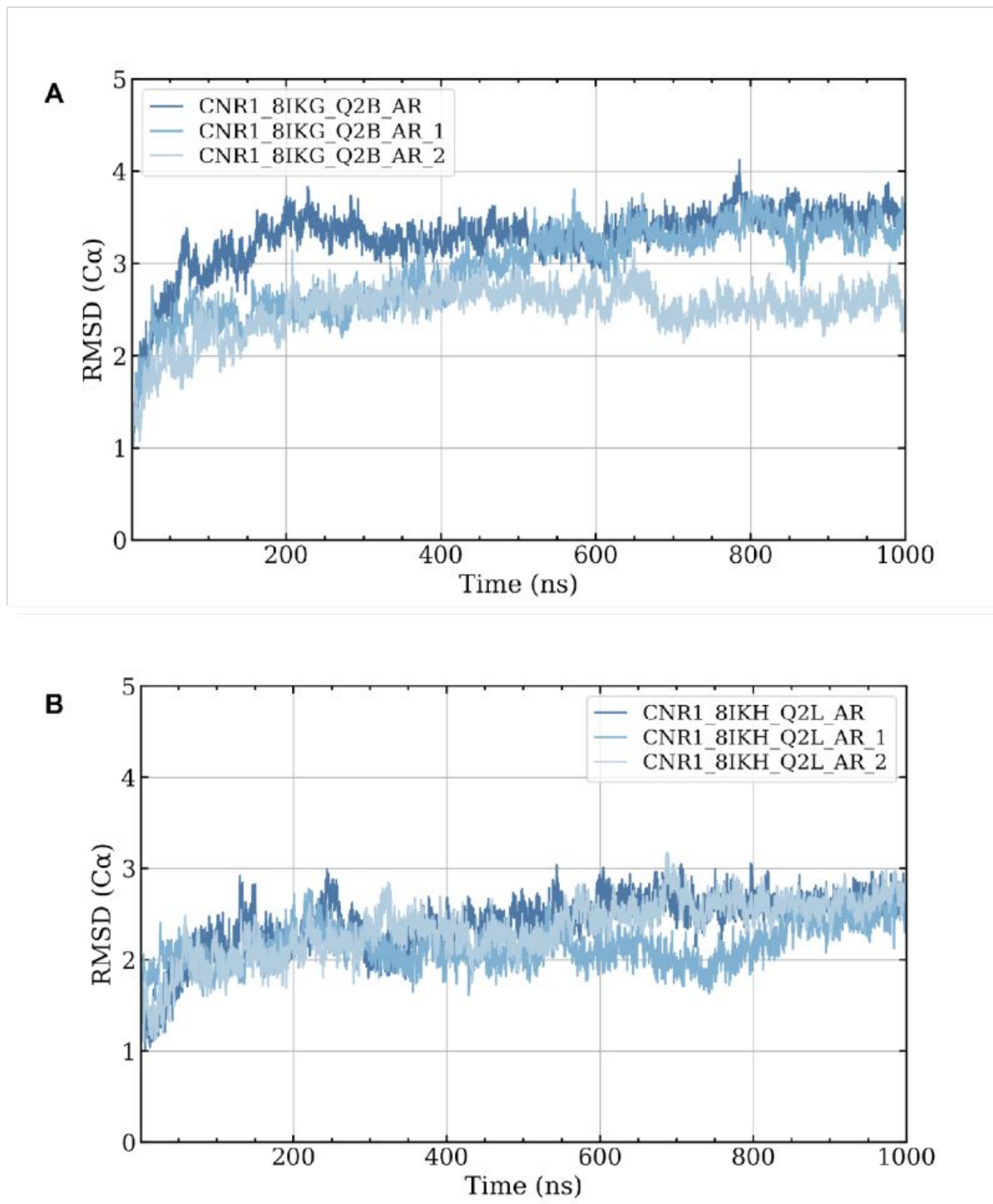

Figure S4. Root Mean Square Deviation (RMSD) of the Cα backbone for two allosteric Gi-biased ligands for Cannabinoid 1 Receptor (CNR1) systems over 1 μs, with three replicas each. (A) RMSD trajectories of the allosteric agonist CB-02-bound structure (PDB: 8IKG) and (B) RMSD trajectories for the allosteric agonist CB-05-bound structure (PDB: 8IKH).

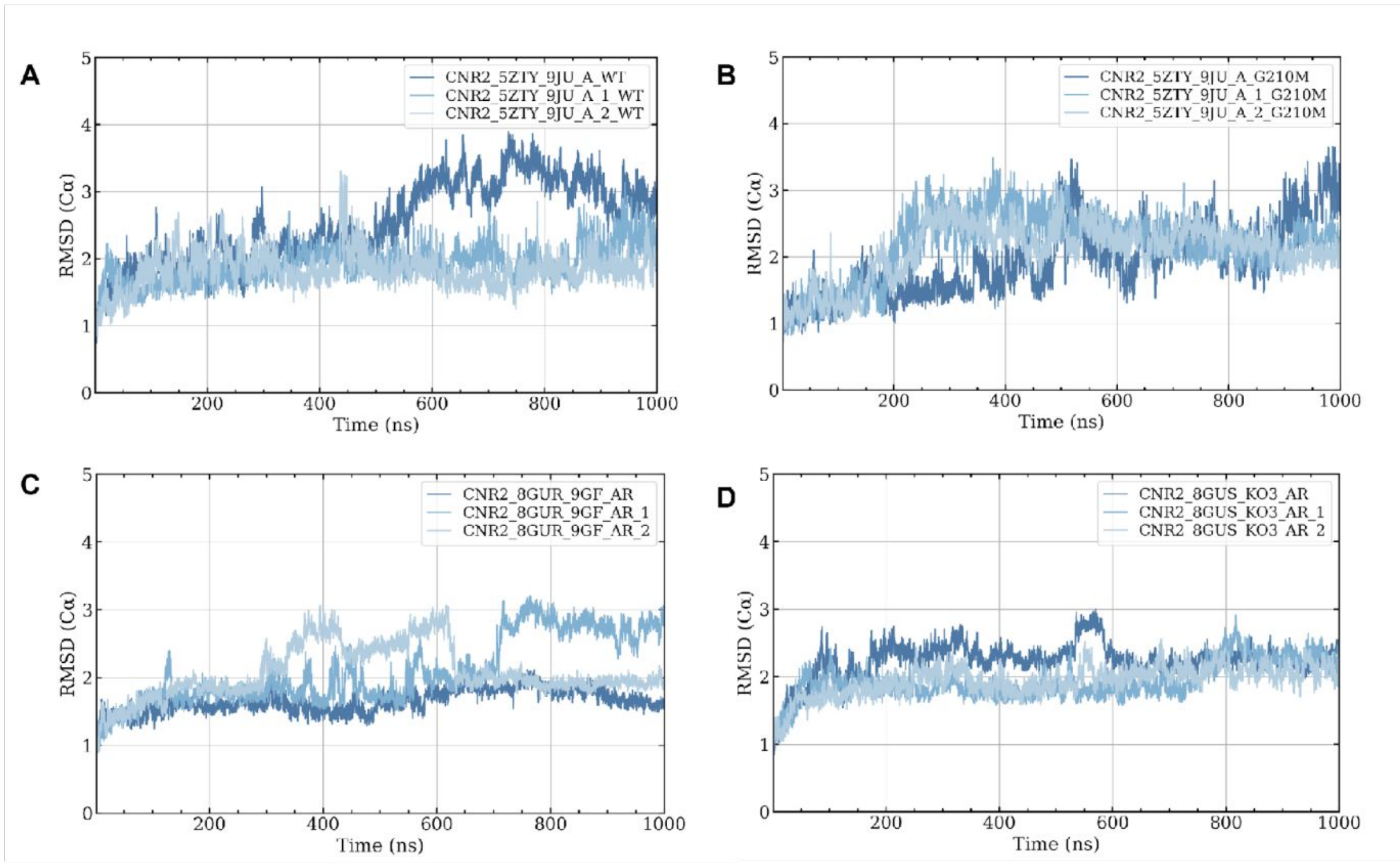

Figure S5. Root Mean Square Deviation (RMSD) of the Cα backbone for Cannabinoid 2 Receptor (CNR2) systems over 1 μs, with three replicas each. (A) RMSD trajectories of the antagonist AM10257 -bound structure (PDB: 5ZTY) and of the same system with G210M mutation, mimicking the corresponding methionine in CNR1. (B) RMSD trajectories for the non-selective agonist CP55,940-bound structure (PDB: 8IKH) and the CNR2-selective agonist HU-308-bound structure (PDB: 8GUS).

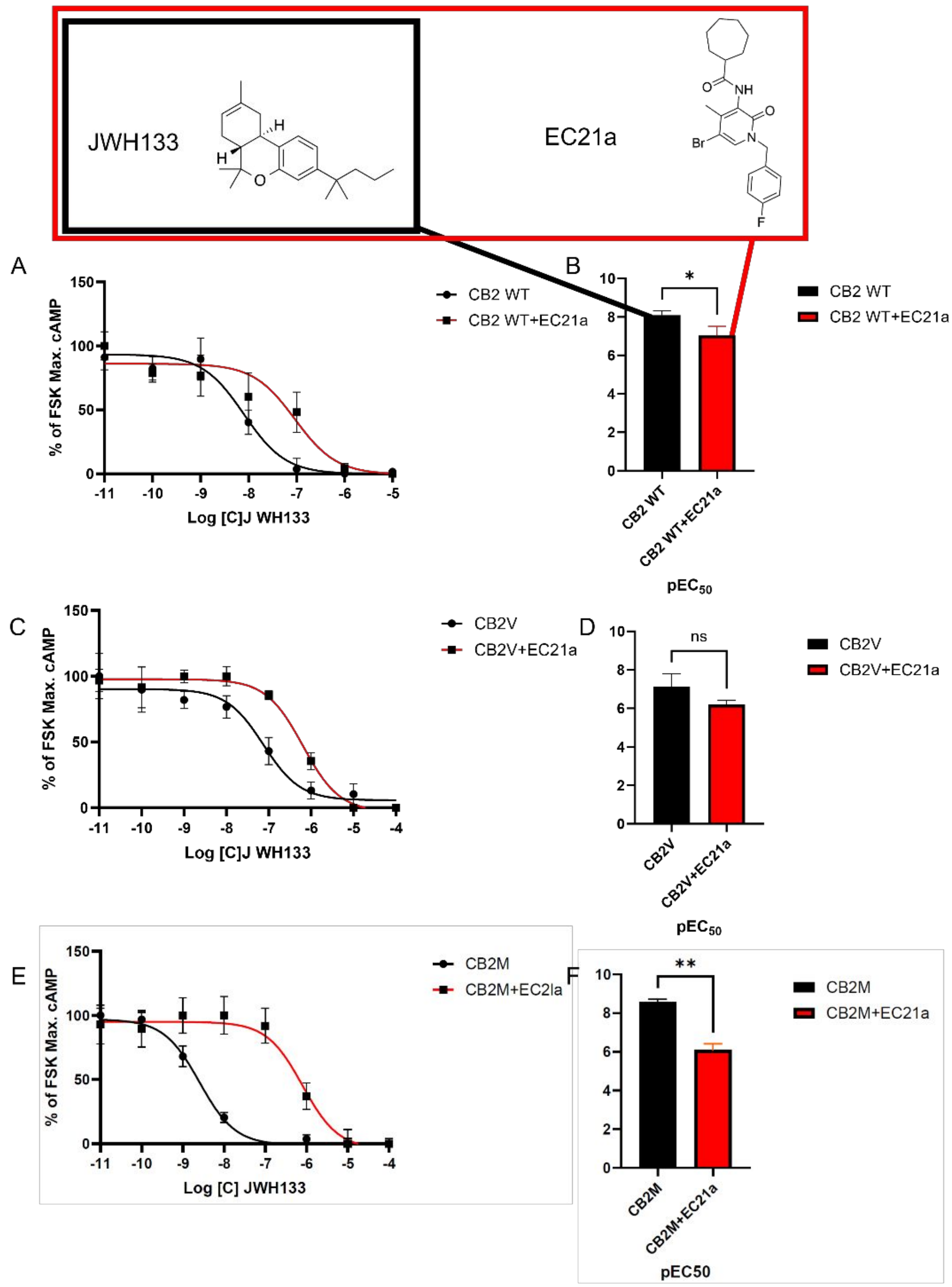

Figure S6. FK-induced cAMP release results for Cannabinoid Receptor 2 (CNR2) WT (A, B), the mutation F197$^{5.46}$V (C, D), G210$^{5.59}$M (E, F). Transfected HEK293T cells were treated with a gradient of concentrations of JWH133 in the presence of absence of the allosteric modulator EC21a. Data shown are the pEC50 values calculated from concentration–response curves. EC50 were statistically compared to JWH133 treatment alone and the ΔpEC50 for each construct were compared to CB2 WT ΔpE_{C50}. Data is presented as mean ± SD from n = 3 independent experiments. $^{*}P < 0.05$; $^{**}P < 0.01$.
